# A comparative morphological revision of the aphid genus *Myzaphis* van der Goot, 1913 (Insecta: Hemiptera: Aphididae) revealed a new genus and three new species

**DOI:** 10.1371/journal.pone.0193775

**Published:** 2018-03-15

**Authors:** Mariusz Kanturski, Shalva Barjadze, Andrew S. Jensen, Karina Wieczorek

**Affiliations:** 1 Department of Zoology, Faculty of Biology and Environmental Protection, University of Silesia, Bankowa 9, Katowice, Poland; 2 Institute of Zoology, Ilia State University, Giorgi Tsereteli 3, Tbilisi, Georgia; 3 Department of Entomology, Washington State University, Pullman, Washington, United States of America; Universita degli Studi della Basilicata, ITALY

## Abstract

The aphid genus *Myzaphis* van der Goot, 1913 from the tribe Macrosiphini is revised to include eight species. Apterous and alate viviparous females, known fundatrices and known sexual morphs (oviparous females and males) of *Myzaphis bucktoni*, *M*. *juchnevitschae*, *M*. *rosarum*, *M*. *tianshanica* and *M*. *turanica* are re-described and illustrated. Lectotype and paralectotypes of *Myzaphis bucktoni* and *M*. *turanica* are designated. The status of *M*. *komatsubarae* nomen dubium is discussed. *Myzaphis avariolosa* is regarded as a species belonging to the genus *Ericaphis*. Three new species: *M*. *oezdemirae* Kanturski & Barjadze **sp. nov.**, *M*. *tuatayae* Kanturski & Barjadze **sp. nov.** from Turkey and *M*. *rezwanii* Kanturski & Barjadze **sp. nov.** from Iran are described and illustrated. *Myzaphis bucktoni* is recorded from Portugal for the first time. Diagnosis of the genus *Myzaphis* van der Goot, 1913 is redefined and a new genus *Richardsaphis* Kanturski & Barjadze **gen. nov.** is erected with the type species *R*. *canadensis* (Richards) **comb. nov.** *Richardsaphis* is for the first time recorded from the USA and hitherto unknown oviparous female and alate male are described and illustrated. Original keys to species of the genus *Myzaphis* and aphid genera of the tribe Macrosiphini with 2-2-2 first tarsal chaetotaxy are also provided.

## Introduction

The aphid genus *Myzaphis* is represented by eight species worldwide [1, 2]. Members of this genus are characterized by a small, elongate-oval body and characteristic rugose or wrinkled dorsal cuticle [3]. They live on undersides of leaves of *Dasiphora* and *Rosa* spp. (Rosaceae), are monoecious holocyclic, rarely anholocyclic and not visited by ants [4]. *Myzaphis rosarum* (Kaltenbach, 1843) and *M*. *turanica* Nevsky, 1929 are pests of the cultivated rose [5]. Five species—*M*. *avariolosa* David, Rajasingh & Narayanan, 1970, *M*. *canadensis* Richards, 1963, *M*. *juchnevitschae* Kadyrbekov, 1993, *M*. *komatsubarae* Shinji, 1922 and *M*. *tianshanica* Kadyrbekov, 1993 are characterized by restricted distribution, while remaining species are distributed widely [4, 6]. Members of the genus *Myzaphis* are morphologically related with species of the genera *Chaetosiphon* Mordvilko, 1914 and *Longicaudus* van der Goot, 1913 and together were in the past regarded as so called “Myzaphidines” [7, 8]. The genus was never reviewed or revised as a whole, besides the “Myzaphidines” or Macrosiphini reviews or new species descriptions on a local scale [7, 8, 9, 10].

Apterous and alate viviparous females of the first species of this genus were described rather precisely from Germany by Kaltenbach [11] as *Aphis rosarum*. Van der Goot [12] erected for *A*. *rosarum* a separate genus–*Myzaphis* on the basis of the unusual characters like body shape, and features of the head and siphunculi. Shinji [6] described the second species from this genus–*M*. *komatsubarae* from Komatsubara Park in Miyakonoyo, Japan. The recorded host plant, *Sorbus commixta*, is unexpected for representatives of this genus and together with very short and poor description [6] calls into question the generic membership of this species [1]. Also, the main problem is the lack of any available material, since Shinji’s collection is considered to be lost and this species was never collected again [9]. The generic placement of a second species, *M*. *canadensis* described from Ontario, Canada, has also been questioned [1]. It varies from other species placed in *Myzaphis* by a few morphological features such as two setae on first tarsal segments, different pattern of the dorsal cuticle, and no dorsal abdominal sclerotic patch in the alate viviparous females given in the original description [7]. The species has only been known from the type series until a recent first report from the United States, where sexual morphs were collected (A. Jensen leg.).

Due to the lack of a genus-level review or revision of *Myzaphis*, the first and second authors’ had opportunity to study *Myzaphis* material held in major collections in Europe, including undetermined material, and the above questions of the monophyly of the group, we decided to undertake a revision of the genus and all the nominal species currently placed in it. This work gave us the opportunity to erect a new genus–*Richardsaphis* Kanturski & Barjadze **gen. nov.** with the type species *R*. *canadensis* (Richards) **comb. nov.,** and describe and illustrate three new species: *M*. *oezdemirae* Kanturski & Barjadze **sp. nov**. and *M*. *tuatayae* Kanturski & Barjadze **sp. nov**. from Turkey and *M*. *rezwanii* Kanturski & Barjadze **sp. nov**. from Iran, living on undetermined *Rosa* spp. In addition, *Myzaphis bucktoni*, *M*. *juchnevitschae*, *M*. *rosarum*, *M*. *tianshanica* and *M*. *turanica* are re-described and illustrated. A paratype of *Myzaphis avariolosa*, a species with large body size and long appendages, was investigated and the species was transferred to the genus *Ericaphis* Börner, 1939.

## Material and methods

### Material

We examined 154 microscopic slides and 474 individuals (36 fundatrices, 316 apterous viviparous females, 47 alate viviparous females, 50 oviparous females and 25 males). The material loaned is deposited in AJ, BMNH, CNC, DZUS, IZKAZ, MNHN, NTPPM, ZMAS, ZMPA, ZMUC. The holotypes and paratypes of the new species are deposited in the MNHN; paratypes of three new species will be also deposited in the Department of Zoology, University of Silesia, Katowice, Poland (DZUS). Host plants names are written originally from slides.

### Scanning Electron Microscopy

Specimens for SEM analyses were preserved in 70% ethanol for several days. For preparation a method modified from that by Kanturski *et al*. [13] was used. From ethanol the specimens were transferred into 6% phosphotungstic acid (PTA) solution in 70% ethanol for 24 hours. Dehydration was provided by ethanol series of 80%, 90%, 96% and two changes of absolute ethanol for 30 minutes each. Dehydrated specimens were dried using hexamethyldisilazane (HMDS) solution with absolute ethanol in proportions of 1:3, 1:2; 2:3 for 30 minutes each followed by two changes of undiluted HMDS. Samples were mounted on aluminium stubs with double-sided adhesive carbon tape and sputter-coated in a Pelco SC-6 sputter coater (Ted Pella Inc., Redding, CA, USA). The specimens were imaged by the Hitachi SU8010 field emission scanning electron microscope FESEM (Hitachi High-Technologies Corporation, Tokyo, Japan) at 5, 10 and 15 kV accelerating voltage with a secondary electron detector (ESD). Sensilla terminology follows Bromley *et al*. [14, 15].

### Light Microscopy

Permanently slide-mounted specimens were imaged using a light Microscope Nikon Eclipse E-600. Photographs were taken by the Nikon DS-Fi2 digital camera with the Nis elements software. Measurements were taken by measuring ocular. Body length (from the median frontal tubercle to the end of cauda) and body parts were measured according to Blackman and Eastop [16]. Digital editing of the figures was made using the PhotoScape v. 3.7 (http://www.photoscape.org/ps/main/index.php). The descriptions terminology follow Blackman and Eastop [16, 3, 4] Kanturski *et al*. [17].

### Abbreviations

The following abbreviations (in the descriptions, re-descriptions, and tables) are used: **BL**–body length; **HW**–head width across compound eyes; **HLS**–head longest seta (on median frontal tubercle or in median area of flat frons); **ANT**–antennae or their lengths; **ANT I**, **II**, **III**, **IV**, **V**–antennal segments I, II, III, IV, V or their lengths; **BASE**–basal part of the last antennal segment or its length; **PT**–terminal process of last antennal segment or its length; **LS III**–longest seta on ANT III; **BD III**–basal articular diameter of ANT III; **URS**–ultimate rostrum segment (IV+V) or its length; **III FEMUR**–hind femur length; **III TIBIA**–hind tibia length; **HT I**–first segment of hind tarsus or its length, **HT II**–second segment of hind tarsus or its length, **SIPH**–siphunculi, **ABD**–abdominal tergite or tergites, **GPL**–genital plate length, **GPW**–genital plate width; **Fx**–fundatrix (stem mother), **apt. viv. fem.**–apterous viviparous female; **al. viv. fem.**–alate viviparous female; **ovip.**–sexual oviparous female; **♂** –male. The collections depository:

**AJ**–Andrew Jensen Aphididae Collection (AphidTrek.org), Lakeview, Oregon, USA.

**BMNH**–The Natural History Museum, London, UK

**CNC–**Canadian National Collection of Insects, Arachnids and Nematodes, Ottawa, Canada

**DZUS**–Department of Zoology, University of Silesia, Katowice, Poland.

**IZKAS**–Institute of Zoology, Kazakhstan Academy of Sciences, Almaty, Kazakhstan.

**MNHN**–Muséum national d’Histoire naturelle, Paris, France.

**NTPPM–**Nazife Tuatay Plant Protection Museum, Ankara, Turkey.

**ZMAS**–Zoological Institute, Russian Academy of Sciences, St. Petersburg, Russia.

**ZMPA**–Zoological Institute, Polish Academy of Sciences, Warsaw, Poland.

**ZMUC–**Zoological Museum, University of Copenhagen, Copenhagen, Denmark.

### Nomenclatural acts

The electronic edition of this article conforms to the requirements of the amended International Code of Zoological Nomenclature, and hence the new names contained herein are available under that Code from the electronic edition of this article. This published work and the nomenclatural acts it contains have been registered in ZooBank, the online registration system for the ICZN. The ZooBank LSIDs (Life Science Identifiers) can be resolved and the associated information viewed through any standard web browser by appending the LSID to the prefix “http://zoobank.org/”. The LSID for this publication is: urn:lsid:zoobank.org:pub:D1CE7CAC-FB12-4C7C-91B0-764CBFF52591. The electronic edition of this work was published in a journal with an ISSN, and has been archived and is available from the following digital repositories: PubMed Central, LOCKSS.

## Results

### *Myzaphis* morphology based on the type species *Myzaphis rosarum*

#### General morphological characters

**Fig 1:**
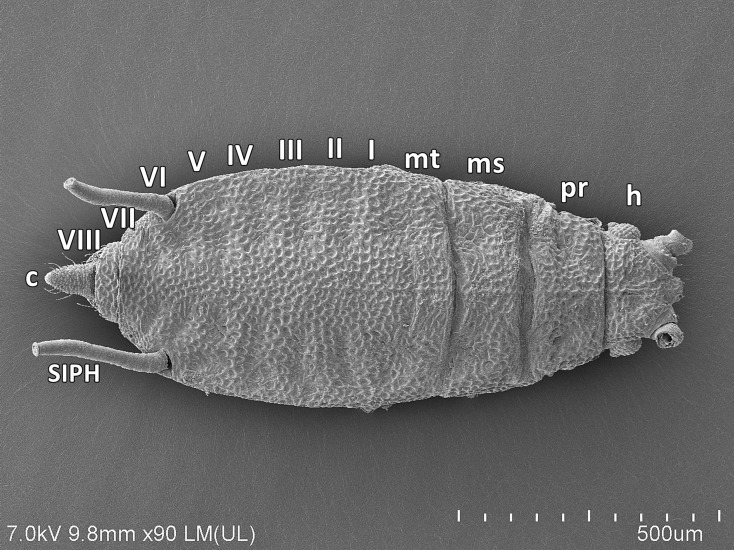
SEM micrograph of apterous viviparous female of *M*. *rosarum* showing the most important features of the genus *Myzaphis*.

**Fig 2:**
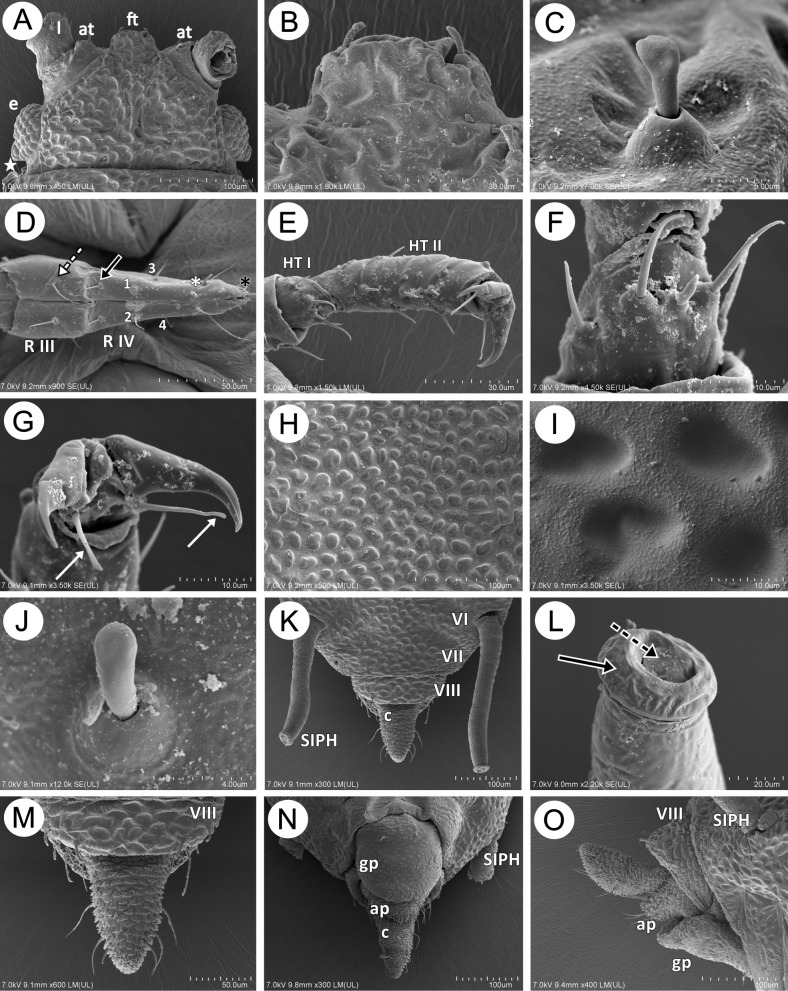
SEM of the most important generic features of the genus *Myzaphis* on the basis of the type species *M*. *rosarum*: A. head showing compound eyes (e), little developed antennal tubercles (at), median frontal tubercle (ft) and ANT I (I). B. shape of median frontal tubercle with two very short setae; C. structure of head seta (trichoid sensillum). D. apical rostrum segments (mouthparts) with type I tricoid sensilla (dotted arrow) on the third segment (R III), fourth segment (R IV) bears pair of type II basiconic sensilla (black arrow), four accessory setae–type I trichoid sensilla (1–4), three pairs of primary setae–type I trichoid sensilla (white asterisk) and type III basiconic sensilla (black asterisk). E. hind tarsus with short first segment (HT I) and longer second segment with claws (HT II). F. ventral side of HT I with five pairs of setae: two pairs of long and fine setae and one central short and rigid “sense peg”. G. apical part of HT II showing normal shaped claws and parempodia with flat apices (white arrows). H. general wiev on the dorsal cuticle. I. rounded or oval recesses on the cuticle; J. structure of dorsal abdominal seta (trichoid sensillum). K. dorsal side of the end of abdomen showing siphunculi (SIPH) on ABD VI (VI), ABD VII (VII) separated from ABD VIII (VIII) and tongue-shaped cauda (c). L. apical part of siphunculus with wide flange (solid arrow) and the siphuncular aperture closed by operculum (dotted arrow). M. ABD VIII (VIII) and cauda with six fine and pointed setae. N. ventral side of the end of abdomen showing the removed siphinculi (SIPH) perianal structures: genital plate (gp), anal plate (ap) and cauda (c). O. lateral side of the end of abdomen showing the siphunculus (SIPH) and the perianal structures: ABD VIII (VIII), cauda, anal plate (ap) and genital plate (gp).

Representatives of the known species of the genus *Myzaphis* are characterized by a narrow, slightly elongated body with evident sculpturing dorsally. The head is separated from pronotum, which is separated from mesonotum. Mesonotum is also separated from the poorly visible or almost invisible metanotum which is fused with ABD I and only a small suture may be noted between those two segments in the spinal area. Almost all abdominal tergites (ABD I-VII) are fused into one more or less sclerotic shield with separate ABD VIII. SIPH are placed on ABD VI. In broad view the body appears hairless (Fig 1). The head is characterized by evident sculpture, more or less developed antennal tubercles and frons (with or without median frontal tubercle). Compound eyes are well-developed but consist of relatively few ommatidia. Triommatidia are also well-developed on small ocular tubercles (Fig 2A). Some species are characterized by a differently shaped median frontal tubercle which can be quadrate or more or less rounded with different numbers of setae. In the case of *M*. *rosarum* the median frontal tubercle is quadrate with two very short setae (Fig 2B). Head setae are very short, robust and rigid, with variously shaped apices. Most head setae are characterized by a well-developed socket, the basal part round and the apex flattened, often wider than the base (Fig 2C). Rostrum is pointed, with few setae: two on segment III, and 12 on segment IV+V, four of which are accessory setae (Fig 2D). Tarsi are characterised by short HT I and HT II with short setae (Fig 2E). On the ventral side of HT I two kinds of setae are visible: four long, hair-like and pointed and one short and rigid central “sense peg” (Fig 2F). The end of HT II is characterized by normal shaped and pointed claws and long parempodia which are rounded from the basal part to about half of their length and then flattened with slightly spatulate apices (Fig 2G). In *M*. *rosarum* the dorsal sculpture seems to be strongly wrinkled (Fig 1) but higher magnification shows it is more or less regular, small, rounded or oval cavities or depressions (Fig 2H and 2I). The dorsum is also characterized by few and inconspicuous setae which are similar to those on the head. Abdominal setae are also very short, rigid, robust or thick with wide, rounded and high sockets, basal part tubular and apices rounded or club-shaped (Fig 2J). The SIPH are more or less straight or slightly curved with the surface imbricated or wrinkled (Fig 2K). Apex of the SIPH with a well-developed, strong flange and well-developed operculum on the siphuncular pore. The SIPH without apical reticulation (Fig 2L). Cauda can be differently developed but always is more or less tongue-shaped, triangular in dorsal and ventral view (Fig 2M and 2N) and oval from lateral side (Fig 2O), with six or seven long, fine and pointed setae. Anal plate and genital plate are covered by fine and pointed setae, but those on genital plate are less numerous and much shorter (Fig 2N and 2O).

#### Sensilla of the genus *Myzaphis*

**Fig 3:**
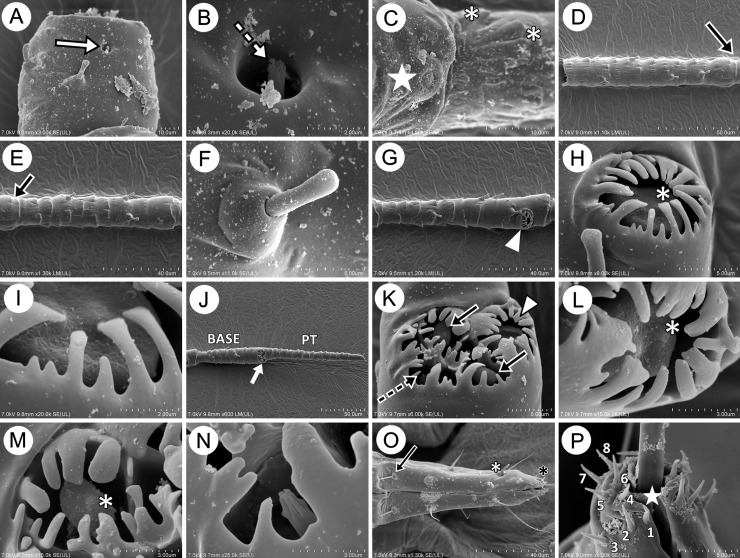
SEM of antennal and mouthparts sensilla of the genus *Myzaphis*: A. very short and rigid type I trichoid sensilla and small rounded rhinariolum (white arrow) on the pedicel. B. structure of the rhinariolum showing sunken peg-like sensillum with 6–8 projections (dotted arrow). C. rounded campaniform sensillum on the pedicel (star) and structure of imbrications on ANT III (asterisks). D. very short, rigid type I trichoid sensilla on ANT III with very scarcely developed border between ANT III and IV (arrow). E. very short, rigid type I trichoid sensilla on ANT III with very scarcely developed border between ANT III and IV (arrow). F. structure of the type I trichoid sensillum on ANT. G. very short and rigid type I trichoid sensilla and big placoid sensillum on ANT V. H. structure of big placoid sensillum on ANT V under the very well developed sclerotic ring. I. structure of the sclerotic ring projections on big placoid sensillum on ANT V. J. type I trichoid sensilla and placoid sensilla on base (BASE) and very short type II trichoid sensilla on the end of processus terminalis (PT) on ANT VI. K. one big placoid sensillum (arrow head), two small placoid sensilla (arrows), and sunken coeloconic sensilla (dotted arrow) on the BASE of ANT VI. L. structure of big placoid sensillum on ANT VI under the very well developed sclerotic ring. M. structure of small placoid sensillum on ANT VI under the very well developed sclerotic ring. N. structure of sunken coeloconic sensillum on ANT VI under the very well developed sclerotic ring. O. URS with type II basiconic sensilla (arrow), type I trichoid sensilla (white asterisk) and type II basiconic sensilla (black asterisk). P. anterior view of the fifth rostral segment showing eight pairs of type III basiconic sensilla around the stylet opening (star).

The antennal sensilla are general divided into campaniform, trichoid, placoid and coeloconic sensilla. On the pedicel, there are two kind of sensilla: single rhinariolum on the ventral side, which is characterised by small (diameter about 2μm) and rounded opening and one peg-like sunken coeloconic sensillum with 6–8 very short projections (Fig 3A and 3B). Dorsal side of the ANT II bears flat and rounded campaniform sensillum with rounded central part (Fig 3C). Surface of antennal segments is covered by clearly visible rings of folds, wrinkles or imbrications that are poorly separated from each other (Fig 3D, 3E and 3G). Antennal segment III and IV are covered only by very few type-I trichoid sensilla which are very similar as those on other body parts. The mentioned sensilla are very short, tubular on the basal part with rounded or club-shaped apices. The sockets are strongly developed, rounded basally and slightly cone-shaped (Fig 3F). On ANT V, near the apex there is a rounded sclerotic ring with very well developed, thick and rigid projections of different length (Fig 3G). The long projections are slightly spatulate and the shorter ones are characterized by more pointed apices. Deep inside the sclerotic ring a big placoid multiporous sensillum is present (Fig 3H and 3I). On ANT VI, on the border between the basal part and the PT there is also a cavity, surrounded by very well-developed sclerotic ring with different shaped projections around and inside. Inside the cavity three kinds of sensilla are present and lie tightly next to each other (Fig 3K). The rounded, big placoid multiporous sensillum is visible under the separate and rounded ring of projections and its structure is the same as in the big placoid sensillum on ANT V (Fig 3L). Near the big placoid sensillum two slightly oval small placoid sensilla can be noted (Fig 3K and 3M). Between and next to the small placoid sensilla there are also 2–3 sunken coeloconic sensilla with 6–7 projections visible (Fig 3K, 3M and 3N).

The mouthparts are covered mainly by trichoid sensilla. URS is covered by three types of sensilla: one pair of fine and pointed type II basiconic sensilla on the proximal part of segment IV, three pair of long, fine and pointed type II trichoid sensilla (Fig 3O) and eight pairs of very short, rigid and pointed type III basiconic sensilla on the poorly separated segment V (Fig 3P).

### Taxonomy

***Myzaphis* van der Goot, 1913**

*Myzaphis* van der Goot, 1913 [12]: 96.

*Francoa* Del Guercio, 1917

Type species: *Aphis rosarum* Kaltenbach, 1843: 101 [11], by original designation

**Diagnosis.** Small, spindle-shaped, or oval aphids with short appendages. Head with weakly developed antennal tubercles, but a characteristic feature of the genus is a strongly projecting quadrate or rounded median tubercle. ANT only about half of body length, without secondary rhinaria in apterae. Alatae have secondary rhinaria on ANT III only, or on ANT III-IV. Dorsal body setae are blunt and somewhat capitate. First tarsal segments all have 5 setae. The dorsum of the aptera is sclerotic and wrinkled or ornamented with numerous small rounded depressions. Alatae have dusky or dark sclerotic markings, often forming a central dorsal abdominal patch. Spiracular apertures are partly covered by opercula. SIPH are elongated, cylindrical for much of their length with the distal part often curved outwards and slightly swollen, and with a small, but distinct flange. The cauda is tongue-shaped or elongated triangular [18].

List of species:

1. ***M*. *bucktoni*** Jacob, 1946
2. ***M*. *juchnevitschae*** Kadyrbekov, 1993
3. ***M*. *komatsubarae*** Shinji, 1922 **nomen dubium**
4. ***M*. *oezdemirae*** Kanturski & Barjadze **sp. nov.**
5. ***M*. *rezwanii*** Kanturski & Barjadze **sp. nov.**
6. ***M*. *rosarum*** (Kaltenbach, 1843) **Type species**
7. ***M*. *tianshanica*** Kadyrbekov, 1993
8. ***M*. *tuatayae*** Kanturski & Barjadze **sp. nov.**
9. ***M*. *turanica*** Nevsky, 1929

Genus ***Ericaphis*** Börner 1939

***E*. *avariolosa*** (David, Rajasingh & Narayanan, 1970) **comb. nov.**

Genus ***Richardsaphis*** Kanturski & Barjadze **gen. nov.**

***R*. *canadensis*** (Richards, 1963) **comb. nov., Type species**

#### Aphid genera of the tribe Macrosiphini with 2-2-2 first tarsal chaetotaxy worldwide

Relatively few genera of Macrosiphini have representatives with only 2 setae on all first tarsal segments, as is the case with the new genus *Richardsaphis*. Consequently it is particularly useful and straightforward to construct the following key to such genera. In the tribe Macrosiphini 2:2:2 first tarsal chaetotaxy is a characteristic feature for the genus *Hydaphias* Börner, 1930 (all five species) on *Galium* spp., subgenus *Galiobium* Börner, 1933 (all two species) of the genus *Myzus* Passerini, 1960 on *Galium* spp., *Brachycaudus (Thuleaphis) acaudatus* (Hille Ris Lambers, 1960) on *Persicaria vivipara*, *Cryptosiphum mordvilkoi* Ivanovskaja, 1960 on *Artemisia* sp., *Hyadaphis haplophylli* Kadyrbekov, 2005 on *Haplophyllum dshungaricum* and *H*. *mongolica* Szelegiewicz, 1969 on *Bupleurum scorzonerifolium*, *Micromyzella kathleenae* Remaudi**è**re, 1985 on *Asplenium praemorsum*, *Pseudacaudella* Börner, 1950 (monotypic genus) on various mosses, and *Staegeriella* Hille Ris Lambers, 1947 (all two species) on *Asperula cynanchica* and *Galium* spp.

Key to aphid genera of the tribe Macrosiphini with 2-2-2 first tarsal chaetotaxy worldwide based on apterous viviparous females

1 ANT III with secondary rhinaria…***Hydaphias*** Börner, 1930
  - ANT III without secondary rhinaria…**2**
2 SIPH pore-shaped and hardly visible; URS stiletto-shaped…***Cryptosiphum*** Buckton, 1879
  - SIPH cylindrical, swollen or truncate, easily visible; URS not stiletto-shaped…**3**
3 Inner faces of antennal tubercles spiculose or scabrous…***Myzus*** Passerini, 1860
  - Inner faces of antennal tubercles smooth…**4**
4 Cauda short and semicircular; SIPH with a marked subapical annular incision…***Brachycaudus*** van der Goot, 1913
  - Cauda triangular or finger-shaped; SIPH without a marked subapical annular incision…**5**
5 Antennal tubercles well developed…***Micromyzella*** Eastop, 1955
Antennal tubercles low or undeveloped…**6**
6 SIPH short, truncate and squamous…***Staegeriella*** Hille Ris Lambers, 1947
  - SIPH long and cylindrical or swollen, not squamous…**7**
7 Dorsum dark pigmented, mainly smooth; SIPH cylindrical…***Pseudacaudella*** Börner, 1950
  - Dorsum pale, membranous and reticulated or wrinkled; SIPH clavate……**8**
8 Dorsum membranous and reticulated; SIPH markedly swollen in the distal half …***Hyadaphis*** Kirkaldy, 1904
  - Dorsum wrinkled; SIPH slightly swollen apically…***Richardsaphis*** Kanturski & Barjadze **gen. nov.**

#### Keys to species of the genus *Myzaphis*

Key to known fundatrices of the genus *Myzaphis*:

1 Dorsal abdominal setae very long (0.02–0.075 mm), thick and pointed…***M*. *tianshanica*** Kadyrbekov
  - Dorsal abdominal setae very short (0.005–0.02 mm), inconspicuous and blunt…**2**
2 SIPH/cauda about 2.69; PT/BASE about 0.66; URS/ANT V about 0.68…***M*. *rezwanii*** Kanturski & Barjadze **sp. nov.**
  - SIPH/cauda 1.96 or less; PT/BASE 0.88 or more; URS/ANT V 0.57 or less…**3**
3 LS/BD III 0.25–0.40; SIPH/cauda 1.65–1.96; URS/ANT V 0.44–0.53 …4
  - LS/BD III 0.57–0.66; SIPH/cauda 1.43–1.51; URS/ANT V 0.54–0.57…***M*. *bucktoni*** Jacob
4 Median frontal tubercle well developed. SIPH and cauda dark …***M*. *rosarum*** (Kaltenbach)
  - Median frontal tubercle absent. SIPH and cauda pale …***M*. *tuatayae*** Kanturski & Barjadze **sp. nov.**

Key to apterous viviparous females of the genus *Myzaphis*:

1 Frons straight or broadly convex without median frontal tubercle…**2**
  - Frons with more or less developed median frontal tubercle that is quadrate or rounded…**3**
2 Dorsum dark; SIPH clavate; ANT V/ANT III 0.57–0.68; Longest head setae 1.25–1.35 x BD III…***M*. *juchnevitschae*** Kadyrbekov
  - Dorsum pale; SIPH not clavate, tapering towards apex; ANT V/ANT III 0.38–0.45; Longest head setae 0.43–0.54 x BD III…***M*. *tuatayae*** Kanturski & Barjadze **sp. nov.**
3 Dorsal body setae long (0.055–0.090 mm), thick and pointed…***M*. *tianshanica*** Kadyrbekov
  - Dorsal body setae very short (0.001–0.025 mm), inconspicuous and blunt … **4**
4 Median frontal tubercle with gentle and delicate edges; almost rounded…**5**
  - Median frontal tubercle clearly quadrate with evident perpendicular edges…**6**
5 Dorsum pale, slightly wrinkled, without dark longitudinal pleural stripes …***M*. *rezwanii*** Kanturski & Barjadze **sp. nov.**
  - Dorsum strongly wrinkled with two broad dark longitudinal pleural stripes …***M*. *bucktoni*** Jacob
6 Median frontal tubercle as long as wide with 2 setae, their length 0.30–0.60 x BD III. Subgenital plate with total 4–7 setae…***M*. *rosarum*** (Kaltenbach)
  - Median frontal tubercle usually wider than long with 2–4 setae, their length 0.80–1.10 x BD III. Subgenital plate with more than 8 setae…**7**
7 URS/ANT III 0.32–0.51; HT II/ANT III 0.42–0.67; Abdomen without two darker longitudinal pleural stripes…***M*. *turanica*** Nevsky
  - URS/ANT III 0.70–0.87; HT II/ANT III 0.85–1.00; Abdomen with two narrow darker longitudinal pleural stripes…***M*. *oezdemirae*** Kanturski & Barjadze **sp. nov.**

Key to known alatae viviparous females of the genus *Myzaphis*:

1 PT/BASE 0.60–0.75; URS/ANT III 0.33–0.34; HT II/ANT III 0.46–0.50…***M*. *juchnevitschae*** Kadyrbekov
  - PT/ BASE 0.95–1.76; URS/ANT III 0.19–0.31; HT II/ANT III 0.26–0.45…**2**
2 ANT V/ANT III 0.61–0.63; HT II/ANT VI 0.30–0.32…***M*. *tianshanica*** Kadyrbekov
  - ANT V/ANT III 0.28–0.51; HT II/ANT VI 0.36–0.72…**3**
3 HW/ANT 0.25–0.30; URS/ANT VI 0.25–0.30; HT II/ANT VI 0.36–0.39; ANT III with 15–24 secondary rhinaria…***M*. *rosarum*** (Kaltenbach)
  - HW/ANT 0.31–0.44; URS/ANT VI 0.34–0.47; HT II/ANT VI 0.44–0.72; ANT III with 6–16 secondary rhinaria…**4**
4 SIPH/cauda 2.00–2.27; HW/ANT 0.43–0.44; ANT IV/ANT III 0.26–0.31; PT/BASE 0.95–1.00…***M*. *rezwanii*** Kanturski & Barjadze **sp. nov.**
  - SIPH/cauda 1.21–1.68; HW/ANT 0.31–0.40; ANT IV/ANT III 0.33–0.46; PT/ BASE 1.05–1.50…**5**
5 ANT IV/ANT III 1.00–1.45…***M*. *bucktoni*** Jacob
  - ANT IV/ANT III 0.31–0.41…***M*. *turanica*** Nevsky

Key to known oviparous females of the genus *Myzaphis*:

1 ANT 5-segmented…**2**
  - ANT 6-segmented…**3**
2 PT/BASE 0.66–1.00; URS/HT II 1.00–1.15; URS/ANT V 0.60–0.83…***M*. *rezwanii*** Kanturski & Barjadze **sp. nov.**
  - PT/BASE 1.20–1.37; URS/HT II 0.70–0.75; URS/ANT VI 0.35–0.36…***M*. *turanica*** Nevsky
3 ANT V/ANT III 0.33–0.40; ANT IV/ANT III 0.33–0.40…***M*. *bucktoni*** Jacob
  - ANT V/ANT III 0.48–0.62; ANT IV/ANT III 0.43–0.57…**4**
4 TIBIAE III with 20–52 pseudosensoria; ANT V/ANT III 0.54–0.62…***M*. *rosarum* (**Kaltenbach)
  - TIBIAE III with 61–80 pseudosensoria; ANT V/ANT III 0.48–0.50…***M*. *oezdemirae*** Kanturski & Barjadze **sp. nov.**

Key to known males of the genus *Myzaphis*:

1 Wings present; ANT IV/ANT III 0.51–0.59…***M*. *oezdemirae*** Kanturski & Barjadze **sp. nov.**
  - Wings absent; ANT IV/ANT III 0.32–0.48…**2**
2 ANT 5-segmented; PT/BASE 0.58–1.37…**3**
  - ANT 6-segmented; PT/BASE 1.42–2.05…**4**
3 PT/BASE 0.58–0.81; ANT V/ANT III 0.62–0.63; URS/ANT V 0.65–0.70…***M*. *rezwanii*** Kanturski & Barjadze **sp. nov.**
  - PT/BASE 1.20–1.37; ANT V/ANT III 0.78–0.86; URS/ANT V 0.26–0.31…***M*. *turanica*** Nevsky
4 ANT III with 17–30, ANT IV with 2–6 secondary rhinaria; ANT/BL 0.70–0.85…***M*. *rosarum*** (Kaltenbach)
  - ANT III with 5–15, ANT IV with 0–1 secondary rhinaria; ANT/BL 0.61–0.64…***M*. *bucktoni*** Jacob

#### Shared characters of representatives of the genus *Myzaphis*

**Fundatrices**. Main characters like in apterous viviparous females. The fundatrices are often larger or smaller than the remaining parthenogenetic and sexual generation. They differ from apterous viviparous females by having 5-segmented antennae. ANT I with 4–7, ANT II with 4–5 setae.

**Apterous viviparous females**. BL 1.04–2.45 mm long. Body shape oval or spindle-shaped. Head with well-developed compound eyes and triommatidia. Antennal tubercles weakly-developed or undeveloped. Frons with median tubercle or flat. Median tubercle or the median area of frons with 2–4 setae, longer or shorter than BD III, blunt or pointed. ANT 6-segmented, without secondary rhinaria. ANT III longest, ANT IV shorter or longer than ANT V. ANT V with one small, rounded primary rhinarium with ciliated edge. ANT VI with one small, rounded primary rhinarium with ciliated edge and 5–6 very small accessory rhinaria, tightly adhering to the primary rhinarium. ANT setae very short, never longer than the width of segments, with blunt apices. ANT I with 5–9, ANT II with 3–6 setae. PT with 3–4 apical setae. Rostrum reaching to middle coxae. Dorsal side of head, thorax and abdomen slightly sclerotized, more or less but evidently wrinkled. Setae on the dorsal side of body extremely short, inconspicuous, with blunt apices. Setae on legs short, never longer than the width of tibiae. First segment of tarsi with 5-5-5 setae. SIPH always longer than cauda, tubular, almost straight, inner side sometimes slightly curved. Cauda tongue-shaped with 6–7 pointed setae.

**Alate viviparous females**. Head and thorax strongly sclerotized. Head with big compound eyes, triommatidia and ocelli. ANT with secondary rhinaria on ANT III and very rarely also on ANT IV. Secondary rhinaria rounded or slightly oval, with smooth edge, arranged irregularly or in 2–3 rows on the whole length of the segment. ANT III longest, ANT IV shorter or longer than ANT V. ANT V with one small, rounded primary rhinarium with ciliated edge. ANT VI with one small, rounded primary rhinarium with ciliated edge and 5–6 very small accessory rhinaria, tightly adhering to the major rhinarium. ANT I with 3–9, ANT II with 3–6 setae. PT with 3–4 apical setae. ANT setae very short, never longer than the width of segments, with blunt apices. Body setae very short and inconspicuous, setae on legs never longer than the width of tibiae. Fore wings hyaline or slightly pigmented (yellow or pale yellow) with brown veins. Media twice branched. Hind wings with two oblique veins. Rostrum reaching to mesosternum. First segment of tarsi with 5-5-5 ventral setae. SIPH always longer than cauda, tubular, slightly swollen from the middle of their length. Cauda tongue-shaped with 6–7 pointed setae.

**Oviparous females**. General characters like in apterous viviparous females. Dorsal side of body much more membranous than in apterous viviparous females, only slightly wrinkled. ANT I with 3–7, ANT II with 4 setae. TIBIAE III much darker than other parts of body, swollen or normal with rounded pseudosensoria (scent plaques) arranged on almost whole length.

**Alate males**. Small apterous (then main morphological features like in apterous viviparous females) and very small alate (then main morphological features like in alate viviparous females). Dorsal side of body more or less sclerotized as irregular sclerites or cross-bars. ANT III-VI with not numerous, small rounded secondary rhinaria like in alate viviparous female. ANT I with 3–7, ANT II with 4–5 setae.

#### Review of species

***Myzaphis bucktoni*** Jacob, 1946

Figs 4A, 5A–8A, 9A, 10A, 11A, 12A, 13A and 14A; Tables 1–5

**Fig 4:**
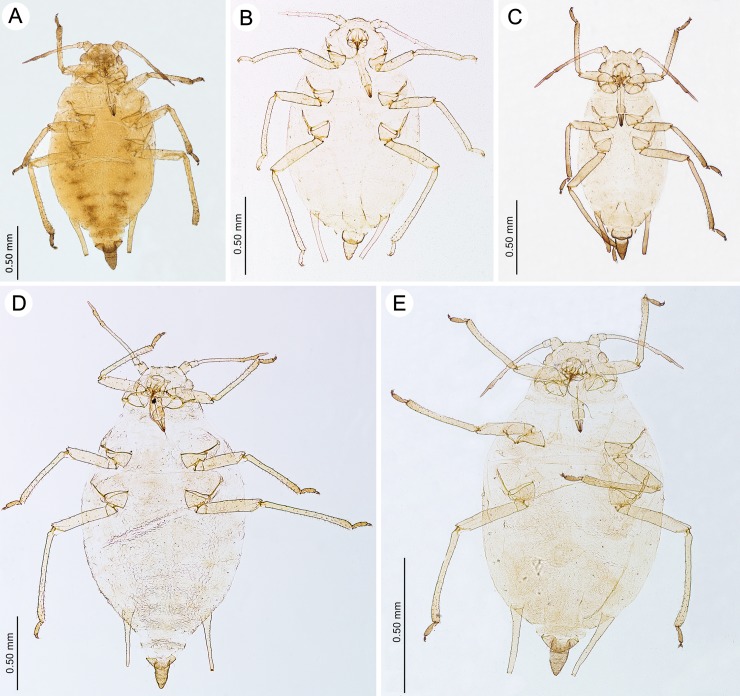
Fundatrices of the genus *Myzaphis*. (A) *M*. *bucktoni*. (B) *M*. *rezwanii* sp. nov. (C) *M*. *rosarum*. (D) *M*. *tianshanica*. (E) *M*. *tuatayae* sp. nov.

**Fig 5:**
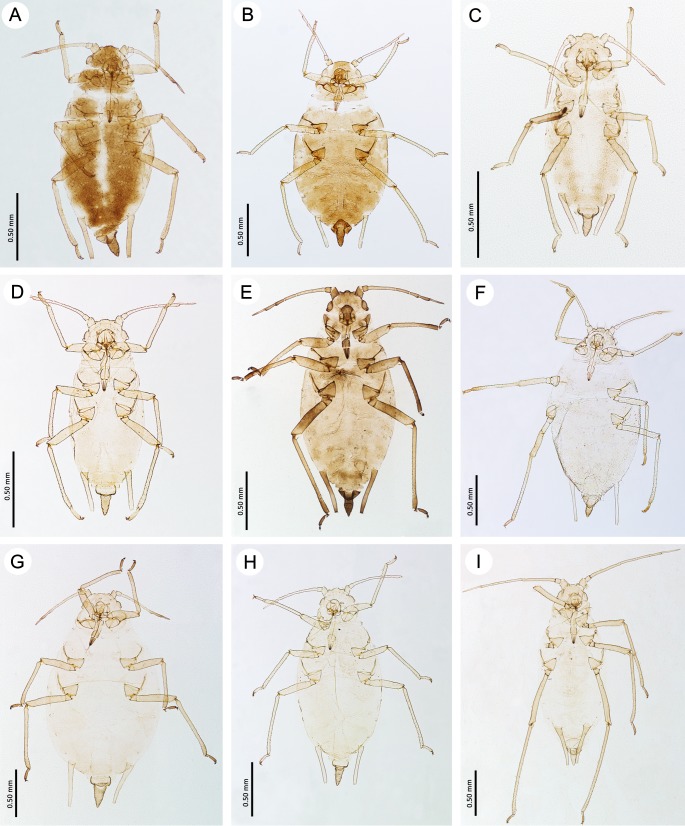
Apterous viviparous females of the genus *Myzaphis* and *Ericaphis*. (A) *M*. *bucktoni*. (B) *M*. *juchnevitschae*. (C) *M*. *oezdemirae* sp. nov. (D) *M*. *rezwanii* sp. nov. (E) *M*. *rosarum*. (F) *M*. *tianshanica*. (G) *M*. *tuatayae* sp. nov. (H) *M*. *turanica*. (I) *E*. *avariolosa* comb. nov.

**Fig 6:**
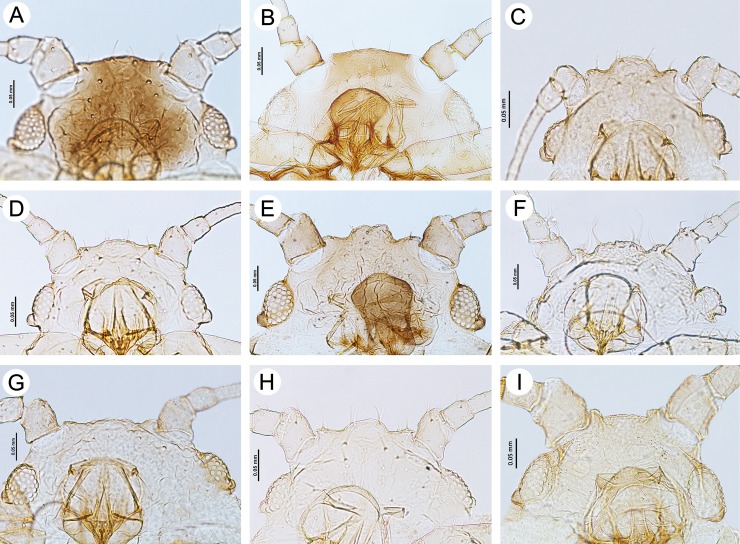
Heads of apterous viviparous females of the genus *Myzaphis* and *Ericaphis*. (A) *M*. *bucktoni*. (B) *M*. *juchnevitschae*. (C) *M*. *oezdemirae* sp. nov. (D) *M*. *rezwanii* sp. nov. (E) *M*. *rosarum*. (F) *M*. *tianshanica*. (G) *M*. *tuatayae* sp. nov. (H) *M*. *turanica*. (I) *E*. *avariolosa* comb. nov.

**Fig 7:**
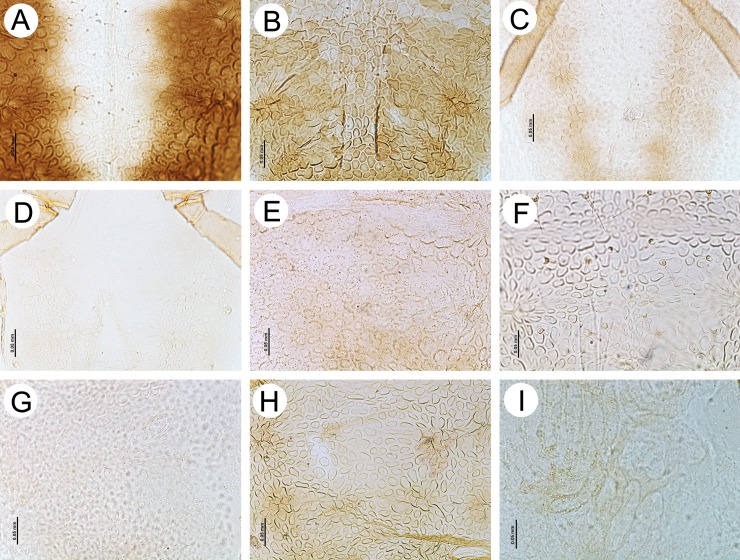
Dorsal abdominal sclerotization patterns of apterous viviparous females of the genus *Myzaphis* and *Ericaphis*. (A) *M*. *bucktoni*. (B) *M*. *juchnevitschae*. (C) *M*. *oezdemirae* sp. nov. (D) *M*. *rezwanii* sp. nov. (E) *M*. *rosarum*. (F) *M*. *tianshanica*. (G) *M*. *tuatayae* sp. nov. (H) *M*. *turanica*. (I) *E*. *avariolosa* comb. nov.

**Fig 8:**
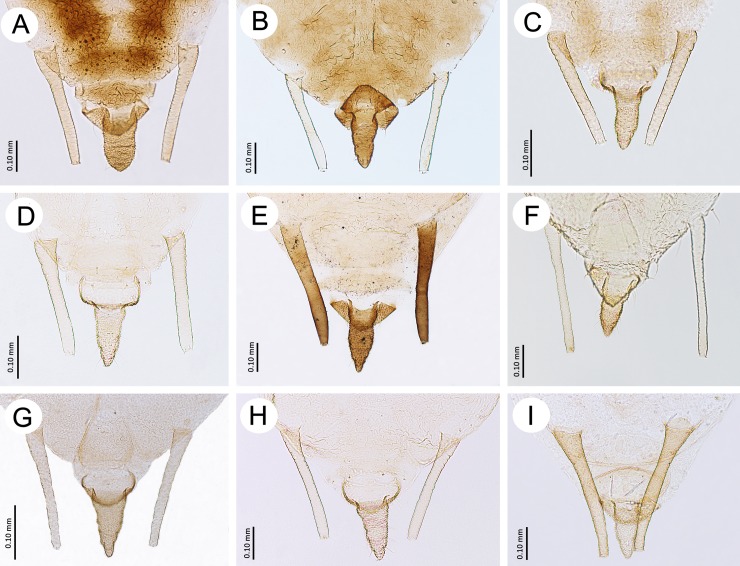
Posterior part of abdomen of apterous viviparous females of the genus *Myzaphis* and *Ericaphis*. (A) *M*. *bucktoni*. (B) *M*. *juchnevitschae*. (C) *M*. *oezdemirae* sp. nov. (D) *M*. *rezwanii* sp. nov. (E) *M*. *rosarum*. (F) *M*. *tianshanica*. (G) *M*. *tuatayae* sp. nov. (H) *M*. *turanica*. (I) *E*. *avariolosa* comb. nov.

**Fig 9:**
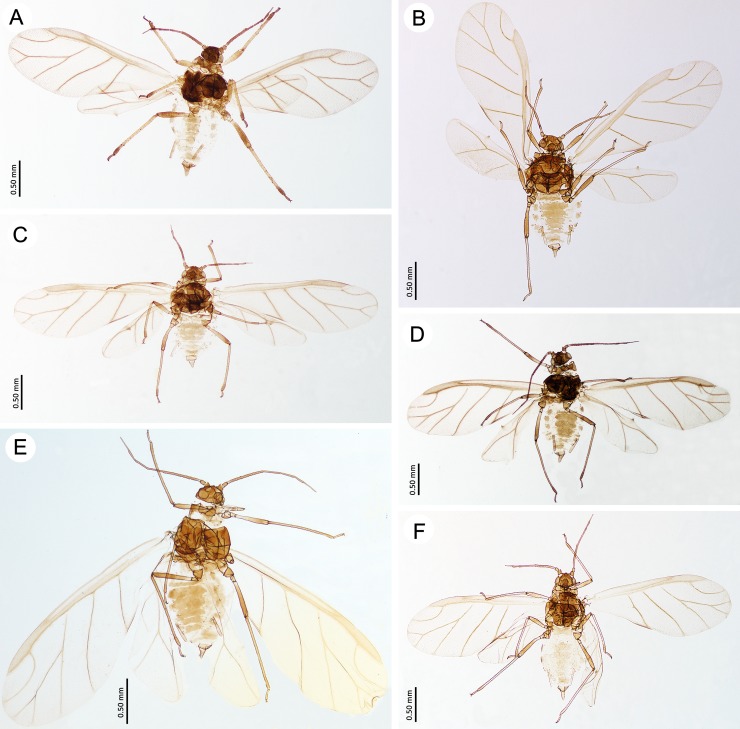
Alate viviparous females of the genus *Myzaphis*. (A) *M*. *bucktoni*. (B) *M*. *juchnevitschae*. (C) *M*. *rezwanii* sp. nov. (D) *M*. *rosarum*. (E) *M*. *tianshanica*. (F) *M*. *turanica*.

**Fig 10:**
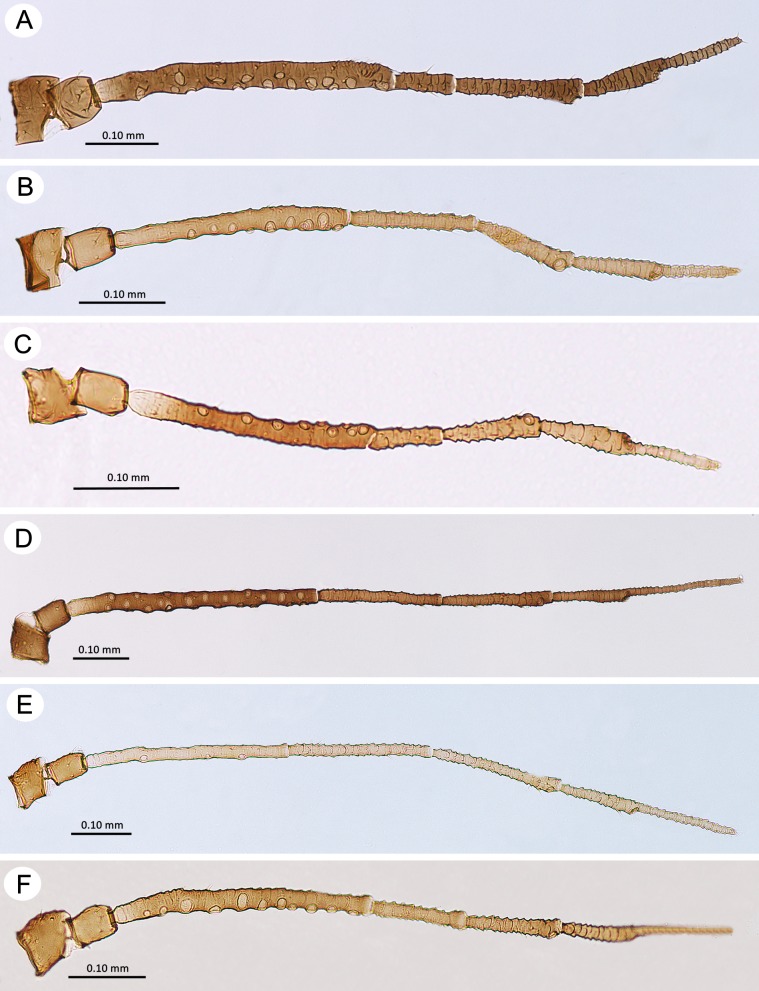
Antennae of alate viviparous females of the genus *Myzaphis*. (A) *M*. *bucktoni*. (B) *M*. *juchnevitschae*. (C) *M*. *rezwanii* sp. nov. (D) *M*. *rosarum*. (E) *M*. *tianshanica*. (F) *M*. *turanica*.

**Fig 11:**
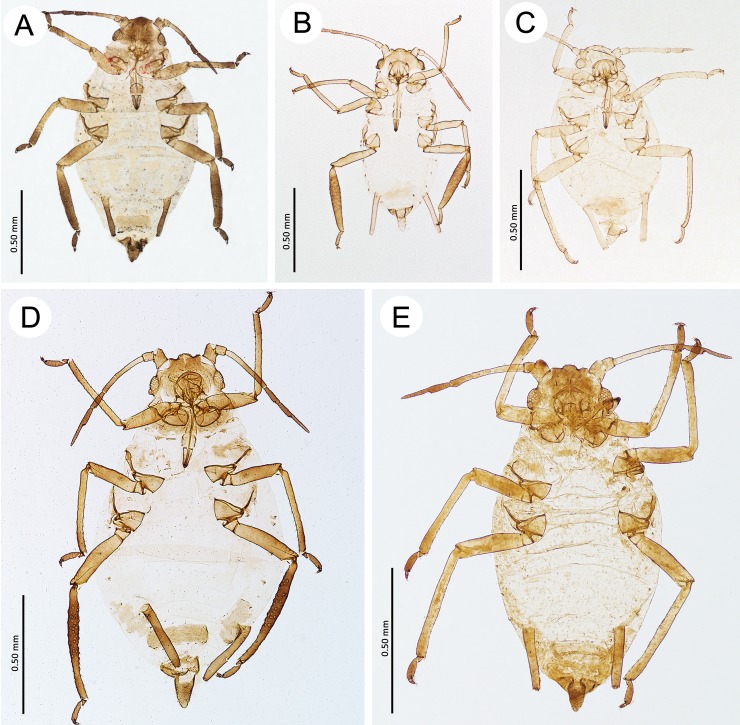
Oviparous females of the genus *Myzaphis*. (A) *M*. *bucktoni*. (B) *M*. *oezdemirae* sp. nov. (C) *M*. *rezwanii* sp. nov. (D) *M*. *rosarum*. (E) *M*. *turanica*.

**Fig 12:**
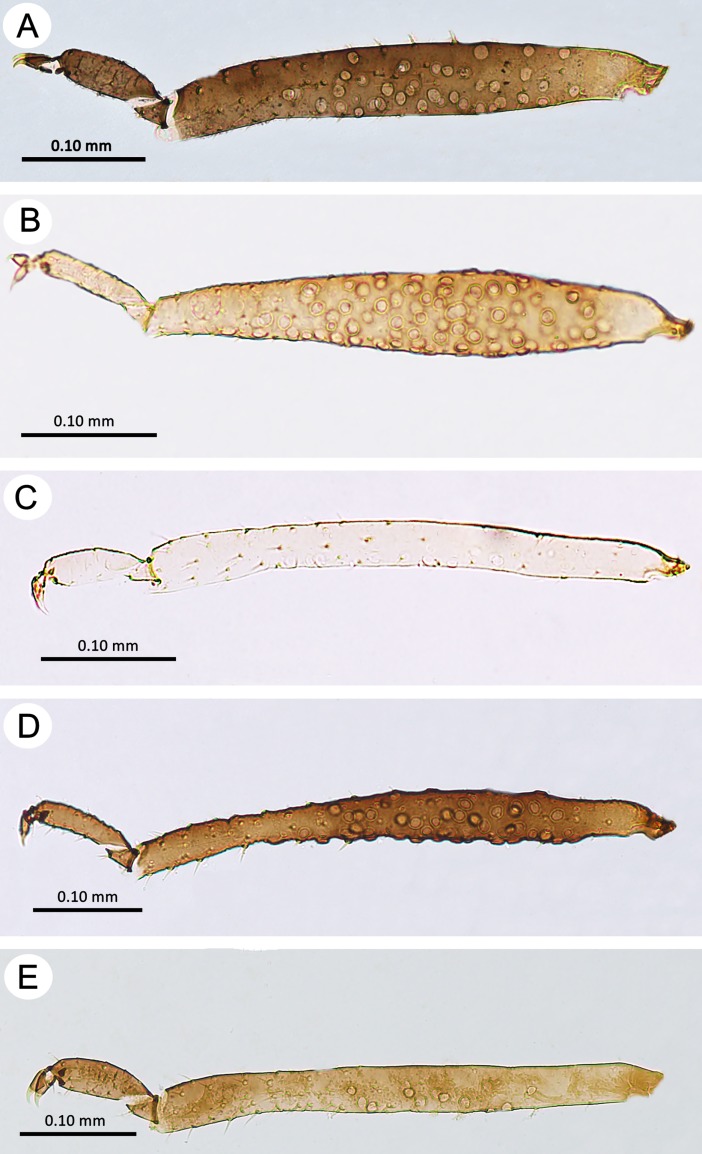
Hind tibiae of oviparous females of the genus *Myzaphis*. (A) *M*. *bucktoni*. (B) *M*. *oezdemirae* sp. nov. (C) *M*. *rezwanii* sp. nov. (D) *M*. *rosarum*. (E) *M*. *turanica*.

**Fig 13:**
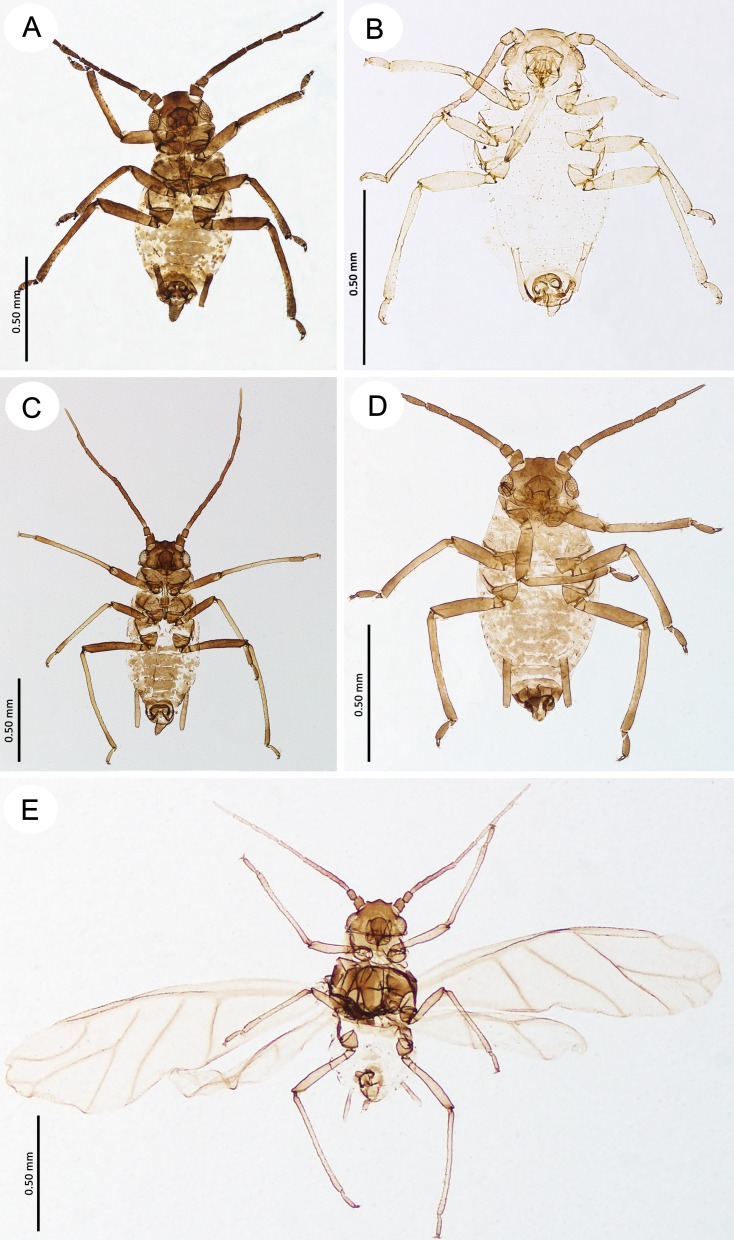
Males of the genus *Myzaphis*. (A) *M*. *bucktoni*. (B) *M*. *oezdemirae* sp. nov. (C) *M*. *rosarum*. (D) *M*. *turanica*. (E) *M*. *rezwanii* sp. nov.

**Fig 14:**
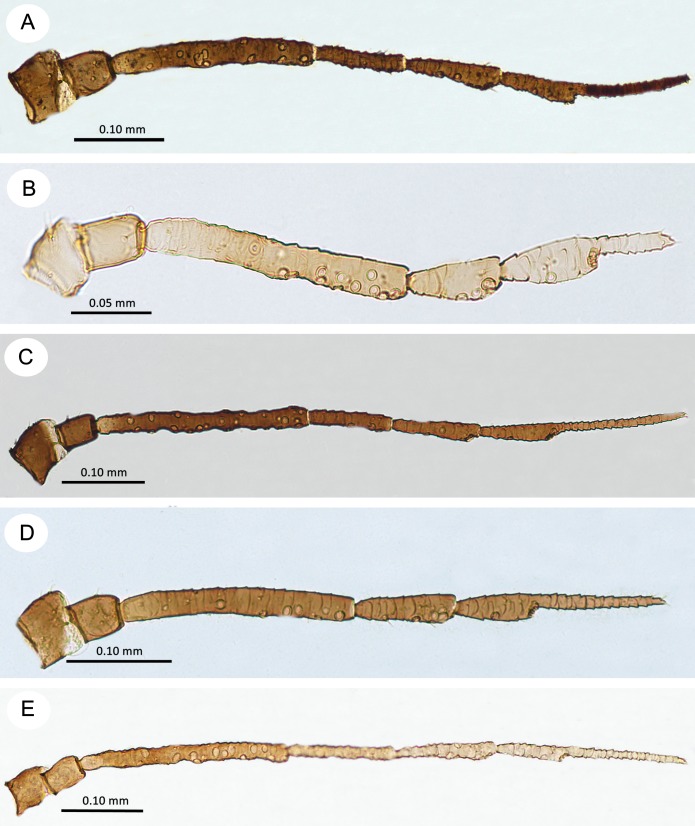
Antennae of males of the genus *Myzaphis*. (A) *M*. *bucktoni*. (B) *M*. *oezdemirae* sp. nov. (C) *M*. *rosarum*. (D) *M*. *turanica*. (E) *M*. *rezwanii* sp. nov.

**Table 1:** Measurements (in mm) of known fundatrices of the genus *Myzaphis*.

Character	*M*. *bucktoni* n = 3	*M*. *rezwanii* n = 21	*M*. *rosarum* n = 10	*M*. *tianshanica* n = 3	*M*. *tuatayae n = 3*
**BL**	2.10–2.60	1.45–1.62	1.10–2.17	2.07–2.25	1.94–2.25
**HW**	0.37–0.43	0.34–0.35	0.28–0.39	0.34–0.38	0.39–0.41
**ANT**	0.60–0.70	0.40–0.47	0.44–0.72	0.58–0.66	0.61–0.62
**ANT III**	0.24–0.27	0.13–0.16	0.15–0.31	0.22–0.25	0.19–0.27
**ANT IV**	0.07–0.10	0.06–0.08	0.06–0.10	0.10–0.12	0.08–0.10
**ANT V**	0.16–0.18	0.10–0.12	0.14–0.19	0.16–0.18	0.17
**BASE**	0.08–0.09	0.06–0.07	0.06–0.10	0.09	0.08–0.09
**PT**	0.08–0.09	0.04–0.05	0.07–0.09	0.07–0.09	0.07–0.08
**URS**	0.09–0.10	0.07–0.08	0.07–0.08	0.08–0.09	0.09–0.10
**III FEMUR**	0.35–0.42	0.28–0.34	0.20–0.36	0.37–0.41	0.40–0.41
**III TIBIA**	0.60–0.68	0.50–0.57	0.37–0.64	0.62–0.70	0.65–0.71
**HT I**	0.03–0.04	0.02–0.03	0.02–0.03	0.03	0.03–0.04
**HT II**	0.10–0.12	0.07–0.08	0.08–0.10	0.10	0.11
**SIPH**	0.31–0.44	0.30–0.35	0.26–0.38	0.39–0.42	0.43–0.46
**CAUDA**	0.22–0.29	0.12–0.13	0.14–0.23	0.19–0.22	0.23–0.25

**Table 2:** Measurements (in mm) of apterous viviparous females of the genus *Myzaphis* and *Ericaphis avariolosa* comb. nov.

Character	*M*. *bucktoni* n = 94	*M*. *juchnevitschae* n = 4	*M*. *oezdemirae* n = 2	*M*. *rezwanii* n = 35	*M*. *rosarum* n = 120	*M*. *tianshanica* n = 2	*M*. *tuatayae* n = 12	*M*. *turanica* n = 41	*E*. *avariolosa* n = 1
**BL**	1.20–2.10	1.52–1.82	1.09–1.13	1.10–1.60	1.04–2.20	1.95–2.00	2.07–2.32	1.65–2.45	2.00
**HW**	0.25–0.38	0.34–0.37	0.25–0.26	0.25–0.32	0.23–0.38	0.34–0.36	0.36–0.40	0.29–0.39	0.35
**ANT**	0.40–0.73	0.50–0.60	0.44	0.48–0.63	0.42–0.89	0.16–0.18	0.66–0.81	0.57–0.92	1.22–1.26
**ANT III**	0.10–0.22	0.12–0.16	0.08–0.10	0.12–0.18	0.09–0.26	0.16–0.18	0.21–0.26	0.15–0.28	0.32–0.38
**ANT IV**	0.04–0.09	0.07–0.08	0.05–0.07	0.05–0.08	0.04–0.14	0.11–0.13	0.08–1.10	0.06–0.14	0.20
**ANT V**	0.05–0.10	0.08–0.09	0.07	0.06–0.09	0.06–0.13	0.10–0.13	0.08–0.12	0.08–0.13	0.17–0.18
**ANT VI**	0.12–0.19	0.14–0.15	0.13–0.14	0.14–0.17	0.15–0.24	0.19–0.22	0.16–0.19	0.16–0.24	0.30–0.35
**BASE**	0.05–0.09	0.08	0.06	0.06–0.07	0.06–0.10	0.09–0.10	0.08–0.09	0.07–0.11	0.10–0.13
**PT**	0.06–0.11	0.05–0.07	0.07–0.08	0.08–0.10	0.08–0.15	0.10–0.12	0.07–0.10	0.09–0.14	0.20–0.22
**URS**	0.07–0.09	0.07	0.07	0.07–0.08	0.047–0.10	0.08	0.08–0.09	0.07–0.10	0.09
**III FEMUR**	0.22–0.41	0.31–0.38	0.21	0.23–0.32	0.19–0.45	0.40–0.42	0.39–0.45	0.31–0.44	0.57–0.58
**III TIBIA**	0.37–0.68	0.50–0.60	0.35	0.38–0.53	0.30–0.79	0.67–0.68	0.50–0.75	0.50–0.74	0.98–0.99
**HT I**	0.02–0.03	0.02–0.03	0.02	0.02–0.03	0.02–0.03	0.03	0.03–0.04	0.02–0.04	0.04
**HT II**	0.08–0.11	0.09–0.10	0.08	0.08–0.10	0.07–0.12	0.10	0.11–0.12	0.09–0.12	0.10–0.11
**SIPH**	0.23–0.42	0.27–0.33	0.23–0.25	0.22–0.31	0.21–0.46	0.41–0.42	0.41–0.44	0.30–0.44	0.42–0.44
**CAUDA**	0.13–0.23	0.16–0.18	0.12–0.14	0.12–0.17	0.13–0.25	0.19–0.20	0.20–0.25	0.16–0.26	0.17–0.19
**GPL**	0.08–0.12	0.10–0.12	0.09	0.08–0.12	0.08–0.15	0.09–0.10	0.12–0.15	0.08–0.14	0.11
**GPW**	0.3–0.20	0.13–0.16	0.14	0.13–0.14	0.17–0.22	0.16–0.17	0.17–0.20	0.15–0.23	0.23

**Table 3:** Measurements (in mm) of known alate viviparous females of the genus *Myzaphis*.

Character	*M*. *bucktoni* n = 13	*M*. *juchnevitschae* n = 1	*M*. *rezwanii* n = 2	*M*. *rosarum* n = 21	*M*. *tianshanica* n = 2	*M*. *turanica* n = 6
**BL**	1.50–1.92	1.62	1.47–1.50	1.85–2.07	2.02	1.87–1.95
**HW**	0.28–0.34	0.31	0.30–0.32	0.34–0.37	0.34	0.30–0.34
**ANT**	0.76–1.02	0.72–0.74	0.67–0.73	1.11–1.35	1.16–1.21	0.84–0.91
**ANT III**	0.27–0.38	0.22	0.24–0.25	0.37–0.46	0.34	0.30–0.34
**ANT IV**	0.12–0.16	0.13	0.06–0.07	0.19–0.22	0.22–0.24	0.10–0.14
**ANT V**	0.10–0.16	0.11–0.12	0.09–0.11	0.17–0.22	0.21	0.10–0.13
**ANT VI**	0.17–0.25	0.16–0.17	0.17–0.20	0.26–0.36	0.27–0.29	0.20–0.21
**BASE**	0.08–0.10	0.10	0.08–0.10	0.11–0.13	0.11–0.13	0.09
**PT**	0.09–0.15	0.06–0.07	0.08–0.10	0.15–0.23	0.16	0.10–0.12
**URS**	0.07–0.09	0.07	0.07	0.08–0.09	0.07	0.08
**III FEMUR**	0.36–0.47	0.39–0.40	0.35–0.37	0.45–0.54	0.48	0.41–0.45
**III TIBIA**	0.63–0.86	0.71–0.73	0.66–0.70	0.86–1.02	0.81–0.83	0.76–0.80
**HT I**	0.02–0.04	0.02–0.03	0.03	0.03	0.02	0.02–0.03
**HT II**	0.10–0.12	0.10–0.11	0.09	0.10–0.13	0.09	0.11–0.15
**SIPH**	0.21–0.26	0.23–0.25	0.25	0.25–0.31	0.27	0.25–0.29
**CAUDA**	0.14–0.19	0.14	0.11–0.12	0.15–0.18	0.14	0.17–0.19
**GPL**	0.09–0.11	0.10	0.07–0.08	0.11–0.13	0.11	0.08–0.12
**GPW**	0.16–0.23	0.16	0.15–0.19	0.18–0.23	0.20	0.17–0.18

**Table 4:** Measurements (in mm) of known oviparous females of the genus *Myzaphis*.

Character	*M*. *bucktoni* n = 13	*M*. *oezdemirae* n = 4	*M*. *rezwanii* n = 4	*M*. *rosarum* n = 19	*M*. *turanica* n = 8
**BL**	1.42–1.52	1.14–1.25	0.94–0.98	1.15–1.67	1.11–1.45
**HW**	0.31–0.32	0.26–0.27	0.23–0.25	0.28–0.32	0.27–0.29
**ANT**	0.50–0.53	0.54–0.56	0.34–0.42	0.59–0.67	0.49–0.59
**ANT III**	0.12–0.13	0.13–0.15	0.12–0.14	0.14–0.22	0.16–0.21
**ANT IV**	0.04–0.05	0.06–0.07	0.05–0.07	0.07–0.09	0.07–0.08
**ANT V**	0.06–0.07	0.07–0.08	0.09–0.12	0.08–0.09	0.16–0.19
**ANT VI**	0.17–0.18	0.16–0.18	–	0.19–0.21	–
**BASE**	0.07–0.08	0.07–0.08	0.04–0.07	0.08–0.09	0.07–0.08
**PT**	0.10	0.08–0.10	0.04–0.06	0.10–0.12	0.09–0.11
**URS**	0.07–0.08	0.07	0.07–0.08	0.07–0.08	0.06–0.07
**III FEMUR**	0.25–0.27	0.24–0.26	0.20–0.23	0.24–0.29	0.23–0.28
**III TIBIA**	0.39–0.42	0.39–0.43	0.33–0.39	0.44–0.50	0.39–0.45
**HT I**	0.03	0.02	0.02	0.03	0.02
**HT II**	0.09–0.10	0.08–0.09	0.06–0.07	0.08–0.10	0.08–0.10
**SIPH**	0.23–0.25	0.23–0.27	0.20–0.23	0.27–0.32	0.21–0.25
**CAUDA**	0.14–0.15	0.13–0.14	0.10–0.11	0.13–0.17	0.10–0.14
**GPL**	0.07–0.08	0.07–0.08	0.08–0.10	0.07–0.10	0.06–0.09
**GPW**	0.18–0.22	0.18–0.20	0.15–0.19	0.20–0.25	0.18–0.21

**Table 5:** Measurements (in mm) of known males of the genus *Myzaphis*.

Character	*M*. *bucktoni* n = 7	*M*. *oezdemirae* n = 3	*M*. *rezwanii* n = 2	*M*. *rosarum* n = 10	*M*. *turanica* n = 2
**BL**	0.95–1.13	0.90–1.25	0.77–0.80	1.02–1.17	0.82–0.95
**HW**	0.27–0.28	0.25–0.27	0.22–0.23	0.27–0.29	0.28
**ANT**	0.60–0.70	0.77–0.87	0.36–0.40	0.78–0.92	0.54–0.58
**ANT III**	0.19–0.21	0.22–0.27	0.15–0.16	0.24–0.28	0.21–0.22
**ANT IV**	0.07–0.09	0.13–0.14	0.05–0.06	0.09–0.13	0.08
**ANT V**	0.07–0.10	0.12–0.13	0.09–0.10	0.11–0.13	0.16–0.19
**ANT VI**	0.17–0.20	0.20–0.24	–	0.22–0.29	–
**BASE**	0.07–0.08	0.08–0.09	0.05–0.06	0.08–0.09	0.07–0.08
**PT**	0.10–0.12	0.12–0.15	0.03–0.04	0.13–0.19	0.09–0.11
**URS**	0.07	0.06–0.07	0.06	0.07	0.05
**III FEMUR**	0.21–0.26	0.26–0.29	0.19–0.20	0.26–0.31	0.21–0.23
**III TIBIA**	0.36–0.46	0.45–0.54	0.35–0.38	0.46–0.58	0.39–0.40
**HT I**	0.02–0.03	0.02–0.03	0.02	0.02–0.03	0.02
**HT II**	0.07–0.09	0.08–0.10	0.06	0.08–0.09	0.07–0.08
**SIPH**	0.15–0.17	0.13–0.15	0.14	0.20–0.22	0.17
**CAUDA**	0.08–0.10	0.08–0.09	0.07	0.10	0.07–0.08

***Myzaphis bucktoni*** Jacob, 1946: 110 [19].

**Material examined. Type material**. **Lectotype** (present designation): UNITED KINGDOM: Wicken Fen Nature Reserve, *Rosa canina*, 20 Jun 1945, F. H. Jacob, apterous viviparous female (apt. viv. fem.) marked by black circle with “L” (BM 1984-264-M1) (BMNH).—Paralectotypes: Wicken Fen Nature Reserve, *R*. *canina*, 20 Jun 1945, F. H. Jacob, apt. viv. fem. (BM 1984-264-M1) (BMNH), 12 apt. viv. fem. (BM 1984-264-M2) (BMNH); Bellingham, Northumberland, *R*. *villosa*, 03 May 1945, F. H. Jacob, fundatrix (fx) (BM 1984-264-M3) (BMNH), fx (BM 1984-264-M4) (BMNH), fx (BM 1984-264-M5) (BMNH); *R*. *villosa*, 03 Nov 1943, F. H. Jacob, 11 oviparous females (ovip.) (BM 1984-264-M6) (BMNH), ovip. (BM 1984-264-M7) (BMNH), ovip. (BM 1984-264-M8) (BMNH); *R*. *villosa*, 03 Nov 1943, F. H. Jacob, apterous male (♂) (BM 1952-236-M9) (BMNH), 6 ♂ (BM 1984-264-M10) (BMNH).

**Non-type material.** AFGHANISTAN: Kabul, plant unknown, 02 Jul 1972, collector unknown, 6 apt. viv. fem. (22812) (MNHN); ALGERIA: IAA, *R*. *rubiginosa*, 20 May 1960, Pasquier, 3 apt. viv. fem. (22816) (MNHN), *Rosa* sp., 11 Jun 1961, Pasquier, alate viviparous female (al. viv. fem.) (22814) (MNHN); ARGENTINA: Lujan de Cuyo, Mendoza, *Rosa* sp. (cult.), 13 Nov 1985, Bahamondes, 2 al. viv. fem. (22817) (MNHN), Mendoza Cd, *Rosa* sp. (cult.), 20 Apr 1986, Bahamondes, 4 apt. viv. fem. (22818) (MNHN), ITALY: Portici, *Rosa* sp., 16 Nov 1938, Roberti, 2 apt. viv. fem. (22809) (MNHN), al. viv. fem. (22815) (MNHN); MEXICO: Calpan Huejotzingo, *Rosa* sp. 05 Jun 1983, Mundz, 4 apt. viv. fem. (22813) (MNHN); MONGOLIA: Ulaan-Bataar, *Rosa* sp., 17 Jul 1963, Szelegiewicz, 7 apt. viv. fem. (R. 2874–1) (ZMPA), 7 apt. viv. fem. (R. 2874–2) (ZMPA), apt. viv. fem. (R. 2874–3) (ZMPA), 2 apt. viv. fem., 2 al. viv. fem. (R. 2874–4) (ZMPA), 3 apt. viv. fem. (R. 2874–5) (ZMPA), 2 apt. viv. fem. (R. 2874–6) (ZMPA), *R*. *acicularia*, 15 Jul 1963, Szelegiewicz, al. viv. fem. (R. 2874–7) (ZMPA); MOROCCO: Rabat, *Rosa* sp. Jun 1928, Mimeur, 2 apt. viv. fem. (22807) (MNHN), 4 apt. viv. fem. (22808) (MNHN); SPAIN: Aranjuez, *Rosa* sp., 07 Jun 1965, Remaudière, 4 apt. viv. fem. (22810) (MNHN), 4 apt. viv. fem. (22811) (MNHN); TURKEY: Ereğli, *Rosa* sp. 18 Jun 1966, Remaudière, 4 apt. viv. fem. (22819) (MNHN), Isparta, *Rosa* sp. 19 May 1961, Tuatay, 2 al. viv. fem. (22820) (MNHN); UNITED KINGDOM: Sutherland, *R*. *canina*, 06 Aug 1972, HLGS, 4 apt. viv. fem. (BM 1982–492) (BMNH); Bangor, *R*. *canina*, 20 Jun 1945, F. H. Jacob, al. viv. fem. (1224) (BMNH); St Mary in the Marsh, Kent, *R*. *canina*, 23 Jun 1950, V. F. Eastop, apt. viv. fem, al. viv. fem. (VFE 7313a) (BMNH), 2 al. viv. fem. (VFE 7313b) (BMNH); USA: Presque Isle, Maine, *R*. *multiflora*, 10 Sep 1956, Simpson & HRL, apt. viv. fem. (BM 1984–340) (BMNH); Presque Isle, Maine, *R*. *palustris*, 14 Aug 1959, O. E. Heie, 6 apt. viv. fem. (2498) (ZMUC).

**Fundatrix–re-description** (n = 3)

(Fig 4A; Table 1)

**Colour.** Colour in life: head dark with paler median part, pronotum pale green with a continuation of the brown head colouring. Abdominal segments have brown markings in the pleural region. Legs pale with tips of tibiae and tarsi brown. Cauda dark brown [19]. Colour in mounted specimens: head yellow to light brown, ANT yellow with apices of ANT IV and V light brown, thorax yellow, abdomen yellow with light brown stripes in the pleural region, more conspicuous at the end. Femora yellow, tibiae yellow with light brown apices and tarsi. (Fig 4A).

**Morphometric characters.** HLS 1.00–1.18 × BD III. ANT 0.26–0.31 × BL and 0.55–0.63 × HW. PT 0.94–1.06 × BASE. Other antennal ratios: ANT V/ANT III about 0.68, ANT IV/ANT III 0.29–0.37. ANT III with 7–9 setae, ANT IV with 3 setae, ANT V with 3 basal setae. LS III 0.57–0.66 × BD III. URS 0.37–0.39 × ANT III, 0.54–0.57 × ANT V, 1.05–1.18 × BASE and 0.83–0.90 × HT II. HT II 0.40–0.44 × ANT III, 1.22–1.31 × BASE and 0.59–0.64 × ANT V. Dorsal setae on thorax 0.015–0.022 mm long; on abdomen 0.017–0.042 mm long. SIPH 1.43–1.51 × cauda.

**Remarks.** Except 5-segmented ANT, this morph differs from the apterous viviparous female by larger BL (2.10–2.60 mm, whereas in the apterous viviparous female BL 1.20–2.10 mm) and lower ratio of SIPH/cauda (1.43–1.51, whereas in apterous viviparous females SIPH/cauda 1.55–1.94).

**Apterous viviparous female–re-description** (n = 94)

(Figs 5A–8A; Table 2)

**Colour.** Colour in life: pale yellow to pale green with light to dark brown dorsal markings in the form of a brown head, two large brown patches of pronotum and a pair of broad pleural stripes extending from the mesonotu almost to the base of cauda [19]. Colour in mounted specimens: ANT, legs and SIPH pale to yellow with brown to dark brown head, pronotum and two broad longitudinal stripes from mesonotum to the end of abdomen, ANT V, ANT VI and cauda (Fig 5A).

**Morphometric characters.** Head with median frontal tubercle rounded, sometimes very low, bearing 4 setae (Fig 6A), HLS 0.90–2.10 × BD III. ANT 0.29–0.41 × BL and 0.44–0.64 × HW. PT 0.93–1.54 × BASE. Other antennal ratios: ANT VI/ANT III 0.88–1.35, ANT V/ANT III 0.41–0.60, ANT IV/ANT III 0.33–0.56. ANT III with 4–9 setae, ANT IV with 2–4 setae, ANT V with 3–4 setae, ANT VI with 3 basal setae. LS III 0.29–0.60 × BD III. URS 0.35–0.70 × ANT III, 0.39–0.57 × ANT VI, 0.83–1.33 × BASE and 0.71–0.94 × HT II. HT II 0.47–0.85 × ANT III, 1.15–1.66 × BASE and 0.39–0.57 ANT VI. Dorsal cuticle mostly in form of squares or pentagons with rounded edges (Fig 7A) Dorsal setae on thorax 0.005–0.050 mm long; on abdomen 0.005–0.031 mm long. SIPH 1.55–1.94 × cauda, almost straight, slightly curved inner side in 1/3 length. Cauda broadly tongue shaped (Fig 8A).

**Alate viviparous female–re-description** (n = 13)

(Figs 9A and 10A; Table 3)

**Colour.** Colour in life: head, ANT and thorax dark brown to black. Legs brown to black. Abdomen pale green with light brown dorsal patch and darker green longitudinal pleural stripes [19]. Colour in mounted specimens: head, ANT and thorax brown to dark brown with lighter pronotum. Fore and hind wings yellowish with brown veins. Pterostigma pale brown with darker edges. Legs yellowish with light brown femora and distal parts of tibiae and tarsi. Abdomen pale with pale brown dorsal sclerotic patch (Fig 9A).

**Morphometric characters**. Head setae 0.007–0.026 mm long, HLS 0.66–1.73 × BD III. ANT 0.46–0.59 × BL and 0.31–0.36 × HW. ANT III with 9–14 secondary rhinaria (Fig 10A). PT 1.05–1.50 × BASE. Other antennal ratios: ANT VI/ANT III 0.58–0.71, ANT V/ANT III 0.28–0.43, ANT IV/ANT III 0.40–0.46. ANT III with 5–10 setae, ANT IV with 3–4 setae, ANT V with 3–5 setae, ANT VI with 3 basal setae. LS III 0.50–0.94 × BD III. URS 0.21–0.29 × ANT III, 0.34–0.47 × ANT VI, 0.76–1.00 × BASE and 0.66–0.81 × HT II. HT II 0.28–0.37 × ANT III, 1.05–1.37 × BASE and 0.46–0.59 × ANT VI. Dorsal setae on pronotum—0.005–0.017 mm long; on abdomen 0.010–0.030 mm long. Dorsal abdominal patch formed from fused sclerites on ABD II-V. SIPH tubular on whole length, 1.21–1.48 × cauda which is broadly tongue-shaped.

**Oviparous female–re-description** (n = 13)

(Figs 11A and 12A; Table 4)

**Colour.** Colour in life: pale olive green, dusky. Head brownish, thorax with two brownish patches on pronotum, ANT, legs, SIPH and cauda dusky translucent. Hind tibiae brown [19]. Colour in mounted specimens: head brown, pronotum light brown, the rest of thorax and abdomen yellow. ANT brown except ANT III. Femora yellow with light brown dorsal parts, fore and middle tibiae yellow with distal parts and tarsi brown. Hind tibiae and tarsi brown to dark brown, SIPH brown with paler basal part, cauda brown (Fig 11A).

**Morphometric characters.** Head with low, rounded median tubercle with 4 setae. Head setae 0.015–0.041 mm long, HLS 1.59–1.60 × BD III. ANT 0.35–0.36 × BL and 0.59–0.62 × HW. PT 1.25–1.50 × BASE. Other antennal ratios: ANT VI/ANT III 1.29–1.36, ANT V/ANT III 0.33–0.40, ANT IV/ANT III 0.30–0.40. LS III 0.48–0.54 × BD III. ANT III with 5–7 setae, ANT IV with 3–4 setae, ANT V with 3–4 setae, ANT VI with 3 basal setae. URS 0.56–0.60 × ANT III 0.44–0.45 × ANT VI, 1.00–1.14 × BASE and 0.80–0.88 × HT II. TIBIAE III conspicuously swollen with 32–46 circular and similar in size pseudosensoria, situated on ¾ length from the proximal part (Fig 12A). HT II 0.66–0.74 × ANT III, 1.25–1.28 × BASE and 0.51–0.55 × ANT VI. Dorsal setae on thorax 0.010–0.020 mm long; on abdomen 0.017–0.040 mm long. SIPH slightly constricted in the middle and slightly swollen at the end, 1.64–1.66 × cauda, which is broadly tongue-shaped.

**Apterous male–re-description** (n = 7)

(Figs 13A and 14A; Table 5)

**Colour.** Colour in life: Abdomen dusky olive green, legs dark brown. All other body parts black [19]. Colour in mounted specimens: Body mostly sclerotized. Head and thorax and sclerotized parts of abdomen brown. ANT dark brown almost black. Legs brown with only slightly paler inner sides. Genitalia, SIPH and cauda dark brown (Fig 13A).

**Morphometric characters.** Head setae 0.012–0.045 mm long, HLS 2.05–4.50 × BD III. ANT (Fig 14A) 0.61–0.64 × BL and 0.39–0.45 × HW. ANT III with 5–15, ANT IV with 0–1, ANT V 2–8 and ANT VI with 0–2 secondary rhinaria. PT 1.42–1.56 × BASE, other antennal ratios: ANT VI/ANT III 0.85–0.97, ANT V/ANT III 0.39–0.47, ANT IV/ANT III 0.37–0.42. ANT III with 8–9 setae, ANT IV with 3–4 setae, ANT V with 3 setae, ANT VI with 3 basal setae. LS III 1.00–1.50 × BD III. URS 0.35–0.39 × ANT III, 0.36–0.44 × ANT VI, 0.93–1.07 × BASE and 0.83–0.93 × HT II. HT II 0.37–0.47 × ANT III, 1.07–1.28 × BASE and 0.43–0.52 ANT VI. Dorsal setae on thorax 0.017–0.020 mm long; on abdomen 0.012–0.030 mm long. SIPH very slightly constricted in the middle and slightly swollen at the end, 1.61–1.93 × cauda, which is broadly tongue-shaped.

**Diagnosis**. Apterous viviparous females of *M*. *bucktoni* differ from other species with little developed and rounded median frontal tubercle by having more or less pigmented longitudinal stripes on the dorsal side of abdomen and longer frontal setae.

**Host plants.** This species lives mostly on *Rosa canina*, *R*. *tomentosa* and *R*. *villosa*. The detailed records for all *Rosa* species are given in Blackman and Eastop [4].

**Distribution.** Widely distributed in Europe except the central (Germany, Poland, Czech Republic, Slovakia, Austria, Switzerland and Hungary) and southern (Balkans) parts. Also in Asia (Afghanistan, Kazakhstan, Mongolia, Pakistan, Turkey), North Africa (Algeria, Morocco), North (USA) and South America (Argentina, Mexico). The detailed records for countries in Palaearctic are given in Holman [20].

***Myzaphis juchnevitchae*** Kadyrbekov, 1993

Figs 5B–8B, 9B and 10B; Tables 2 and 3

*Myzaphis juchnevitchae* Kadyrbekov, 1993: 105 [10].

**Material examined. Paratypes**. KAZAKHSTAN: mount Ketmen, 10 km SE of Bol’shoy Aksu, *Rosa albertii*, 23 Jun 1987, R. Kadyrbekov, 2 apt. viv. fem. (22802) (MNHN), 13 Jun 1987, 2 apt. viv. fem., al. viv. fem. (692) (IZKAS).

**Apterous viviparous female–re-description** (n = 4)

(Figs 5B–8B; Table 2)

**Colour.** Colour in life: brown with black cauda [10]. Colour in mounted specimens: Body evidently sclerotized, yellow to light brown, ANT, legs and SIPH yellowish, cauda brown (Fig 5B).

**Morphometric characters.** Front of head broadly convex in dorsal view, with no distinct median frontal tubercle and two slightly pointed setae (Fig 6B), 0.010–0.027 mm long. HLS 1.25–1.35 × BD III. ANT 0.30–0.33 × BL and 0.61–0.67 × HW. PT 0.64–0.82 × BASE. Other antennal ratios: ANT VI/ANT III 0.93–1.12, ANT V/ANT III 0.57–0.68, ANT IV/ANT III 0.51–0.60. ANT III with 6–7 setae, ANT IV with 3–4 setae, ANT V with 3 setae, ANT VI with 2 basal setae. LS III 0.50–0.60 × BD III. URS 0.45–0.57 × ANT III, 0.48–0.51 × ANT VI, 0.82–0.93 × BASE and 0.75–0.77 × HT II. HT II 0.60–0.76 × ANT III, 1.05–1.25 × BASE and 0.64–0.68 ANT VI. Dorsal cuticle mostly in the form of squares, diamonds or polygons with very rounded edges that tightly adjoin to each other (Fig 7B). Dorsal setae on thorax 0.007–0.012 mm long; on abdomen 0.010–0.030 mm long. SIPH 1.54–1.88 × cauda, clavate from about 1/3 length. Cauda tongue shaped (Fig 8B).

**Alate viviparous female–re-description** (n = 1)

(Figs 9B and 10B; Table 3)

**Colour.** Colour in life: head, thorax and dorsal abdominal patch brown. Rest of abdomen and SIPH greenish yellow, cauda black [10]. Colour in mounted specimens: head, ANT and thorax brown. Fore and hind wings pale with brown veins. Pterostigma pale brown with slightly darker edges. Legs with light brown femora and yellowish tibiae with darker distal parts and tarsi. Abdomen pale with light brown dorsal sclerotic patch, marginal sclerites, SIPH and cauda (Fig 9B).

**Morphometric characters.** Head setae 0.012–0.015 mm long, HLS about 1.00 × BD III. ANT (Fig 14C) 0.44–0.45 × BL and 0.41–0.43 × HW. ANT III with 10–11 secondary rhinaria (Fig 10B). PT 0.60–0.75 × BASE. Other antennal ratios: ANT VI/ANT III 0.72–0.72, ANT V/ANT III 0.52–0.53, ANT IV/ANT III 0.57–0.63. ANT III with 7 setae, ANT IV with 3 setae, ANT V with 4 setae, ANT VI with 2 basal setae. LS III about 0.66 × BD III. URS 0.33–0.34 × ANT III, 0.42–0.46 × ANT VI, about 0.75 × BASE and 0.68–0.71 × HT II. HT II 0.46–0.50 × ANT III, 1.05–1.10 × BASE and 0.60–0.68 × ANT VI. Dorsal setae on pronotum 0.007–0.010 mm long; on abdomen 0.010–0.040 mm long. Dorsal abdominal patch formed from fused sclerites on ABD I-VI. SIPH clavate, 1.64–1.78 × cauda which is narrow tongue-shaped.

**Diagnosis.** Among all known species of *Myzaphis* only *M*. *juchnevitchae* and *M*. *tuatayae* Kanturski & Barjadze **sp. nov.** are characterized by completely flat frons without median frontal tubercle. *M*. *juchnevitchae* can be distinguished from the new species by (1) the length of the setae on head, (2) darker pigmentation of abdomen, and (3) clavate SIPH.

**Host plants.** It was collected from *Rosa albertii*.

**Distribution.** The species is known only from the Ketmen Mountains and Tyshkan in Borokhoro (Tien Shan) in Kazakhstan.

***Myzaphis oezdemirae*** Kanturski & Barjadze **sp. nov.**

urn:lsid:zoobank.org:act:5149AE05-9F9F-488F-B72E-07829A2CBDB5

Figs 5C–8C, 11B, 12B, 13E and 14E; Tables 2, 4 and 5

as *Myzaphis turanica* Nevsky, 1929 in Tuatay & Remaudière, 1964: 264–265 [21].

**Material examined. Holotype**. TURKEY: Tatvan, *Rosa* sp., 09 Oct 1962, Remaudière, apt. viv. fem. (22785) (MNHN).

**Paratypes**. TURKEY: Tatvan, *Rosa* sp., 09 Oct 1962, Remaudière, apt. viv. fem. (Tur62/10/1) (DZUS), ovip. (22793) (MNHN), ovip. (22794) (MNHN), ovip. (22795) (MNHN), al. ♂ (22791) (MNHN), al. ♂ (22792) (MNHN); Bitlis, *Rosa* sp., 11 Oct 1962, Remaudière, ovip. (22796) (MNHN); IRAN: Gatch e Sar, 1900m, *Rosa* sp. 09 Nov 1967, Remaudière, al. ♂ (22790) (MNHN).

**Apterous viviparous female–description** (n = 2)

(Figs 5C–8C; Table 2)

**Colour.** Colour in life: unknown. Colour in mounted specimens: body in general pale brown or yellowish with slightly darker narrow longitudinal stripes in pleural region of abdomen (Fig 5C).

**Morphometric characters.** Head with large, rectangle-shaped median frontal tubercle, bearing 4 setae (Fig 6C). Head setae 0.006–0.025, HLS 1.35–1.66 × BD III. ANT 0.38–0.40 × BL and 0.56–0.59 × HW. PT 1.25–1.33 × BASE. Other antennal ratios: ANT VI/ANT III 1.35–1.75, ANT V/ANT III 0.70–0.87, ANT IV/ANT III 0.55–0.93. ANT III with 3–4 setae, ANT IV with 3 setae, ANT V with 3 setae, ANT VI with 2–3 basal setae. LS III 0.29–0.33 × BD III. URS 0.70–0.87 × ANT III, 0.50–0.51 × ANT VI, about 1.16 × BASE and 0.82–0.87 × HT II. HT II 0.85–1.00 × ANT III, 1.33–1.41 × BASE and 0.57–0.62 ANT VI. Dorsal cuticle mostly in form of rectangles with rounded edges or oval (Fig 7C). Dorsal setae on thorax 0.006–0.075 mm long; on abdomen 0.006–0.025 mm long. SIPH 1.78–1.91 × cauda, straight, not clavate. Cauda tongue shaped (Fig 8C).

**Oviparous female–re-description** (n = 4)

(Figs 11B and 12B; Table 4)

**Colour.** Colour in life: unknown. Colour in mounted specimens: head, ANT IV-VI, fore and middle legs, hind femora yellow to pale brown. Hind tibiae brown. The rest of body pale or yellowish (Fig 11B).

**Morphometric characters.** Head with low, rounded median tubercle with 4 setae. Head setae 0.005–0.020, HLS 0.85–1.00 × BD III. ANT 0.44–0.47 × BL and 0.46–0.50 × HW. PT 1.02–1.25 × BASE. Other antennal ratios: ANT VI/ANT III 1.10–1.33, ANT V/ANT III 0.48–0.50, ANT IV/ ANT III 0.48–0.50. LS III 0.35–0.37 × BD III. ANT III with 4–6 setae, ANT IV with 3 setae, ANT V with 3 setae, ANT VI with 3 basal setae. URS 0.50–0.55 × ANT III 0.41–0.45 × ANT VI, about 1.16 × BASE and 0.77–0.88 × HT II. TIBIAE III strongly swollen with 61–80 circular and similar in size pseudosensoria, situated on almost whole length of tibiae (Fig 12B). HT II 0.60–0.64 × ANT III, 1.06–1.20 × BASE and 0.47–0.54 × ANT VI. Dorsal setae on thorax 0.005–0.007 mm long; on abdomen 0.005–0.025 mm long. SIPH almost straight, inconspicuously clavate, 1.67–2.07 × cauda, which is tongue-shaped.

**Alate male–re-description** (n = 3)

(Figs 13E and 14E; Table 5)

**Colour.** Colour in life: unknown. Colour in mounted specimens: Head and thorax sclerotized, brown. ANT III and genitalia light brown. The rest of ANT segments, legs, abdominal patch and SIPH pale to yellow. Fore and hind wings pale yellow with light brown veins. Pterostigma pale brown with very slightly darker edges (Fig 13E).

**Morphometric characters.** Head setae 0.007–0.017 mm long, HLS 1.00–1.41 × BD III. ANT (Fig 14E) 0.70–0.86 × BL and 0.30–0.32 × HW. ANT III with 13–19, ANT IV without, ANT V with 4–7 and ANT VI with 0–2 secondary rhinaria. PT 1.41–1.70 × BASE, other antennal ratios: ANT VI/ANT III 0.90–0.93, ANT V/ANT III 0.48–0.59, ANT IV/ ANT III 0.51–0.59. ANT III with 5–8 setae, ANT IV with 3–5 setae, ANT V with 3 setae, ANT VI with 3 basal setae. LS III 0.66–0.83 × BD III. URS 0.25–0.27 × ANT III, 0.28–0.29 × ANT VI, 0.70–0.76 × BASE and 0.65–0.73 × HT II. HT II 0.35–0.40 × ANT III, 1.00–1.17 × BASE and 0.38–0.43 ANT VI. Dorsal setae on pronotum 0.007–0.010 mm long; on abdomen 0.005–0.020 mm long. SIPH slightly clavate, constricted near base, 1.66–1.93 × cauda, which is tongue-shaped.

**Remarks.** We re-describe here the sexuales, because the first brief descriptions of these morphs were given by Tuatay and Remaudière [20] as sexual morphs of *M*. *turanica*.

**Etymology.** The authors have the pleasure in naming the new species to honour our colleague Dr. Isil Özdemir, aphid taxonomist in Turkey.

**Diagnosis.** Three species of *Myzaphis* (*M*. *rosarum*, *M*. *turanica* and *M*. *oezdemirae* **sp. nov.**) are characterized by having a distinctly developed, square-shaped or rectangular frontal median tubercle with short, inconspicuous, and blunt setae in apterous viviparous females. Both *M*. *turanica* and *M*. *oezdemirae* **sp. nov.** differ from *M*. *rosarum* by frontal tubercle wider than long with longer setae 0.80–1.10 x BD III (square-shaped frontal tubercle with shorter setae 0.30–0.60 x BD III in *M*. *rosarum*). Apterous viviparous females of *M*. *oezdemirae* **sp. nov.** differ from *M*. *turanica* by: (1) pigmented abdomen with two narrow, light brown longitudinal pleural stripes (abdomen pale without pigmentation in *M*. *turanica*), (2) higher ratio of URS/ANT III, 0.70–0.87 (0.32–0.51 in *M*. *turanica*), (3) higher ratio of HT II/ANT III, 0.85–1.00 (0.42–0.67 in *M*. *turanica*) and (4) higher ratio of ANT VI/ANT III, 1.35–1.75 (0.86–1.19 in *M*. *turanica*). Oviparous females differ by 6-segmented ANT and numerous pseudosensoria (61–80) on strongly swollen hind tibiae (5-segmented ANT, 7–20 pseudosensoria on normal shaped hind tibiae in *M*. *turanica*). The males differ first of all by being alate with 6-segmented ANT and 13–19 secondary rhinaria on ANT III (apterous, with 5-segmented antennae and 7–11 secondary rhinaria on ANT III in *M*. *turanica*).

**Host plants.** It was collected from an unidentified *Rosa* species.

**Distribution.** The new species is known from Tatvan and Bitlis in eastern Turkey and Gatch e Sar in Iran.

***Myzaphis rezwanii*** Kanturski & Barjadze **sp. nov.**

urn:lsid:zoobank.org:act:B7FF7D7C-630A-4B23-A200-7A4DF1BBC639

Figs 4B, 5D–8D, 9C–10C, 11C, 12C, 13B and 14B; Tables 1–5

**Material examined. Holotype**. IRAN: Lalezar, Kerman, *Rosa* sp., 26 Jun 1955, Remaudière, apt. viv. fem. number 1 in red circle with annotation “H” on the slide (22766) (MNHN). **Paratypes**. IRAN: Lalezar, Kerman, *Rosa* sp., 26 Jun 1955, Remaudière, 3 apt. viv. fem. number 2, 3, 4 (22766) (MNHN), 6 apt. viv. fem. (Irn55/06/1) (DZUS), 4 apt. viv. fem. (22762) (MNHN), 2 apt. viv. fem. (22764) (MNHN), 4 apt. viv. fem. (22767) (MNHN), 24 Jun 1955, Remaudière, apt. viv. fem. (22767) (MNHN); Rte Gatch e Sar, *Rosa* sp., 16 Apr 1963, Remaudière, 4 fx (22757) (MNHN), 4 fx (22759) (MNHN), 4 fx (22760) (MNHN), 4 fx (22761) (MNHN); 30 km S from Dargaz, *Rosa* sp., 18 May 1966, Remaudière, al. viv. fem. (22768) (MNHN), al. viv. fem. (22769) (MNHN), 2 fx (22770) (MNHN), 3 fx (22771) (MNHN); Pai qual Eh, *Rosa* sp., 28 Jun 1955, Remaudière, 2 apt. viv. fem. (22763) (MNHN); Ahar, *Rosa* sp., 05 Jun 1964, Remaudière, 4 apt. viv. fem. (22772) (MNHN), 4 apt. viv. fem. (22780) (MNHN), 4 apt. viv. fem. (22781) (MNHN); Fachand, *Rosa* sp., 21 Oct 1955, Remaudière, ovip. (22775) (MNHN), ovip. (22776) (MNHN), ovip. (22777) (MNHN), ovip. (22778) (MNHN), ♂ (22773) (MNHN), ♂ (22774) (MNHN).

**Fundatrix–description** (n = 21)

(Fig 4B; Table 1)

**Colour.** Colour in life: unknown. Colour in mounted specimens: all body parts pale to yellowish. (Fig 4B).

**Morphometric characters.** HLS 0.90–1.60 × BD III. ANT 0.27–0.29 × BL and 0.71–0.75 × HW. PT 0.66–0.83 × BASE. Other antennal ratios: ANT V/ANT III about 0.78–0.80, ANT IV/ANT III about 0.50. ANT III with 4–8 setae, ANT IV with 3–4 setae, ANT V with 2 basal setae. LS III 0.40–0.60 × BD III. URS 0.53–0.57 × ANT III, 0.68–0.72 × ANT V, 1.13–1.33 × BASE and 0.94–1.0 × HT II. HT II 0.56–0.57 × ANT III, 1.20–1.33 × BASE and 0.71–0.72 × ANT V. Dorsal setae on thorax 0.010–0.017 mm long; on abdomen 0.012–0.037 mm long. SIPH 2.50–2.69 × cauda.

**Remarks:** Besides 5-segmented ANT, this morph differs from apterous viviparous female by often wider body, shorter ANT– 0.40–0.47 mm long (0.48–0.63 mm in apterae), higher ratio HW/ANT– 0.71–0.85 (0.50–0.56 in apterae) and lower ratio PT/BASE– 0.66–0.83 (1.20–1.50 in apterae).

**Apterous viviparous female–description** (n = 35)

(Figs 5D–8D; Table 2)

**Colour.** Colour in life: unknown. Colour in mounted specimens: all body parts pale yellow, with slightly darker cauda (Fig 5D). In some specimens, also ANT VI and tarsi darker.

**Morphometric characters.** Head with median frontal tubercle rounded, sometimes very low, bearing 4 setae (Fig 6D). Head setae 0.007–0.032 mm long, HLS 0.90–1.60 × BD III. ANT 0.34–0.43 × BL and 0.50–0.56 × HW. PT 1.20–1.50 × BASE. Other antennal ratios: ANT VI/ANT III 0.94–1.29, ANT V/ANT III 0.42–0.57, ANT IV/ANT III 0.36–0.51. ANT III with 4–7 setae, ANT IV with 3–4 setae, ANT V with 2–5 setae, ANT VI with 3 basal setae. LS III 0.45–0.50 × BD III. URS 0.43–0.66 × ANT III, 0.42–0.53 × ANT VI, 1.00–1.33 × BASE and 0.70–0.94 × HT II. HT II 0.56–0.75 × ANT III, 1.28–1.53 × BASE and 0.56–0.62 ANT VI. Dorsal cuticle mostly in form of ovals or more or less irregular circles, little developed (Fig 7D). Dorsal setae on thorax 0.005–0.012 mm long; on abdomen 0.005–0.025 mm long. SIPH 1.61–2.00 × cauda, straight or only slightly curved, very rarely only slightly clavate near the apex. Cauda tongue shaped (Fig 8D).

**Alate viviparous female–description** (n = 2)

(Figs 9C and 10C; Table 3)

**Colour.** Colour in life: unknown. Colour on mounted specimens: head, ANT and thorax brown. Fore and hind wings yellowish with brown veins. Pterostigma pale brown with darker edges. Legs pale brown with distal parts of femora, tibiae and tarsi. Abdomen pale with dorsal sclerotic patch, SIPH and cauda pale brown (Fig 9C).

**Morphometric characters.** Head setae 0.010–0.020 mm long, HLS 1.33–1.70 × BD III. ANT 0.45–0.48 × BL, and 0.43–0.44 × HW. ANT III with 8–110 secondary rhinaria (Fig 10C). PT 0.95–1.00 × BASE. Other antennal ratios: ANT VI/ANT III 0.70–0.82, ANT V/ANT III 0.37–0.44, ANT IV/ANT III 0.26–0.31. ANT III with 5–8 setae, ANT IV with 3 setae, ANT V with 3 setae, ANT VI with 2 basal setae. LS III 0.80–1.20 × BD III. URS 0.28–0.31 × ANT III, 0.34–0.44 × ANT VI, 0.66–0.88 × BASE and 0.76–0.83 × HT II. HT II 0.36–0.37 × ANT III, 0.87–1.05 × BASE and 0.44–0.52 × ANT VI. Dorsal setae on pronotum 0.010–0.015 mm long; on abdomen 0.010–0.047 mm long. Dorsal abdominal patch formed from fused, wide sclerites on ABD II-IV and narrower sclerites on ABD V and VI. SIPH distinctly clavate, constricted near base, 2.00–2.27 × cauda which is broadly tongue-shaped with broad basal part.

**Oviparous female–description** (n = 4)

(Figs 11C and 12C; Table 4)

**Colour.** Colour in life: unknown. Colour in mounted specimens: all body parts pale yellow to pale brown (Fig 11C).

**Morphometric characters.** Head with very poorly developed frontal median tubercle with four setae. Head setae 0.007–0.022 mm long, HLS 0.88–1.46 × BD III. ANT 5-segmented, 0.35–0.44 × BL and 0.58–0.67 × HW. PT 0.66–1.00 × BASE. Other antennal ratios: ANT V/ANT III 0.72–0.91, ANT IV/ANT III 0.44–0.50. LS III 0.58–0.66 × BD III. ANT III with 3–8 setae, ANT IV with 2–3 setae, ANT V with 2 basal setae. URS 0.53–0.62 × ANT III 0.60–0.83 × ANT VI, 1.06–1.66 × BASE and 1.00–1.15 × HT II. TIBIAE III not swollen with 4–10 scarcely visible, circular and similar in size pseudosensoria, situated on the inner side in the middle of tibiae (Fig 12C). HT II 0.51–0.54 × ANT III, 0.96–1.44 × BASE and 0.57–0.72 × ANT V. Dorsal setae on thorax 0.007–0.012 mm long; on abdomen 0.005–0.045 mm long. SIPH clavate, slightly constricted in the middle, 2.00–2.09 × cauda, which is broadly tongue-shaped.

**Apterous male–description** (n = 2)

(Figs 13B and 14B; Table 5)

**Colour.** Colour in life: unknown. Colour in mounted specimens: head, ANT and genitalia pale brown. The rest of body pale to pale yellow (Fig 13B).

**Morphometric characters.** Head setae 0.010–0.020 mm long, HLS about 1.20 × BD III. ANT 5-segmented 0.45–0.51 × BL and 0.57–0.60 × HW. ANT III with 10–12, ANT IV with 3–4, ANT V 1–2 secondary rhinaria (Fig 14B). PT 0.58–0.81 × BASE. Other antennal ratios: ANT V/ANT III 0.62–0.63, ANT IV/ANT III 0.33–0.37. ANT III with 4–5 setae, ANT IV with 2–3 setae, ANT V with 3 basal setae. LS III about 1.20 × BD III. URS 0.40–0.44 × ANT III, 0.65–0.70 × ANT V, 1.11–1.18 × BASE and about 1.08 × HT II. HT II 0.37–0.41 × ANT III, 1.03–1.09 × BASE and 0.60–0.65 ANT V. Dorsal setae on thorax 0.07–0.010 mm long; on abdomen 0.005–0.035 mm long. SIPH clavate, constricted in the middle, about 2.00 × cauda, which is tongue-shaped with broad medial part.

**Etymology.** The authors have the pleasure in naming the new species to honour Dr. A. Rezwani, who worked on the Iranian aphid fauna for several decades.

**Diagnosis.** Together with *M*. *bucktoni* the new species differs from other *Myzaphis* species by rounded frontal median tubercle which is sometimes very low. Apterous viviparous females of *M*. *rezwanii* **sp. nov.** differ from *M*. *bucktoni* by (1) unpigmented body without brown longitudinal pleural stripes (body pigmented with two brown longitudinal pleural stripes in *M*. *bucktoni*) and (2) poorly sclerotized cuticle (strongly sclerotized in *M*. *bucktoni*). Alate viviparous females of *M*. *rezwanii* **sp. nov.** differ from those of *M*. *bucktoni* by: (1) higher ratio of HW/ANT– 0.43–0.44 (0.31–0.36 in *M*. *bucktoni*), (2) lower ratio PT/BASE– 0.95–1.00 (1.05 extremely rarely, usually 1.15–1.50 in *M*. *bucktoni*), (3) lower ratio ANT IV/ANT III– 0.26–0.31 (0.40–0.46 in *M*. *bucktoni*), (4) higher ratio of SIPH/cauda– 2.00–2.27 (1.21–1.48 in *M*. *bucktoni*). Oviparous females of the new species differ from those of *M*. *bucktoni* by: (1) 5-segmented ANT (6-segmented in *M*. *bucktoni*), (2) lower ratio PT/BASE– 0.66–1.00 (1.25–1.50 in *M*. *bucktoni*), (3) higher ratio of URS/HT II– 1.00–1.15 (0.80–0.88 in *M*. *bucktoni*), (4) higher ratio of SIPH/cauda– 2.00–2.09 (1.64–1.66 in *M*. *bucktoni*) and (5) lower number of pseudosensoria on TIBIAE III– 4–10 (32–46 in *M*. *bucktoni*). Males of the new species differ from those of *M*. *bucktoni* by (1) 5-segmented ANT (6-segmented in *M*. *bucktoni*), (2) lower ratio of PT/BASE– 0.58–0.81 (1.42–1.56 in *M*. *bucktoni*) and (3) pale body with membranous abdomen (body dark pigmented with sclerotized abdomen in *M*. *bucktoni*).

**Host plants.** It was collected from unidentified wild *Rosa* species.

**Distribution.** The species is known from several montane localities (1300–2750 a. s. l.) in Iran.

***Myzaphis rosarum*** (Kaltenbach, 1843)

Figs 4C, 5E–8E, 9D, 10D, 11D, 12D, 13C and 14C; Tables 1–5

*Aphis rosarum* Kaltenbach, 1843: 101 [11].

*Francoa elegans* Del Guercio, 1917

*Trilobaphis rhodolestes* Wood-Baker, 1943

*Aphis rossrum* Raychaudhuri, Ghosh & Das, 1980

**Material examined.** Because Kaltenbach did not designate type material of aphids he described, no name-bearing type specimen is believed to be extant. The authors consider that a name-bearing type is necessary to define the nominal taxon objectively. According to the International Code of Zoological Nomenclature (Article 75.1), the Neotype of *M*. *rosarum* is here designated: **Neotype** (present designation): FRANCE: Samoëns, *Rosa* sp. 21 May 1977, Remaudière, apt. viv. fem. in red circle with “N”, 3 apt. viv. fem (22837) (MNHN).

**Non-type material.** AFGHANISTAN: Kabul, host plant not known, 27 Jun 1972, collector unknown, 6 apt. viv. fem. (22826) (MNHN); ALGERIA: Alger plage, *Rosa* sp., 17 May 1960, Pasquier, 6 apt. viv. fem. (22833) (MNHN); 2 al. viv. fem. (22835) (MNHN); CHILE: Mulchen (Bio-Bio), *Rosa* sp., 09 Nov 1974, HRL, al. viv. fem. (BM 1984–340), 7 apt. viv. fem. (BM 1984–340) (BMNH); CZECH REPUBLIC: Trencin, plant unknown, 30 May 1964, Eastop., al. viv. fem. (VFE 10 052) (BMNH); DENMARK: Tarm, *Dasyphora fruticosa*, 19 Oct 1991, O. E. Heie, apt. viv. fem. (8151) (ZMUC); FAROE ISLANDS: Thorshavn, *Rosa* sp., 29 Aug 1968, O. E. Heie, apt. viv. fem. (3480) (ZMUC); FINLAND: Helsinki, *Rosa rugosa*, 25 Jun 1963, O. E. Heie, 6 apt. viv. fem. (3480ab) (ZMUC); FRANCE: Vars, *Rosa* sp. (pubescent), 20 Jun 1972, Remaudière, 2 fx (22842) (MNHN), fx (22843) (MNHN), 4 fx (22844) (MNHN); Utelle, *Rosa* sp., 11 May 1969, Remaudière & Leclant, fx (22841) (MNHN), St. Guilhem, *Rosa* sp., 15 Jun 1967, Remaudière & Leclant, 2 fx (22855) (MNHN); La Varenne, *Rosa* sp., 07 Jun 1957, Remaudière, 5 apt. viv. fem. (22840) (MNHN), 25 Jun 1989, Remaudière, 4 apt. viv. fem. (22822) (MNHN), 24 Jul 1983, Leclant, 4 apt. viv. fem. (22831) (MNHN), 20 May 1962, Remaudière, 2 al. viv. fem. (22854) (MNHN), 25 Apr 1989, Remaudière, 2 al. viv. fem. (22823) (MNHN), Jardin des Plantes, *Rosa* sp., 01 Jun 1994, Remaudière, 2 apt. viv. fem. (22821) (MNHN), Samoëns, *Rosa* sp. 21 May 1977, Remaudière, 2 al. viv. fem (22836) (MNHN), 2 al. viv. fem. (22838) (MNHN), Risoul, *Rosa* sp. 22 Aug 1987, Remaudiére, 3 apt. viv. fem. (22839) (MNHN), La Combe, *R*. *canina* 14 Oct 1978, Remaudière, 4 ovip. (22849) (MNHN), ♂ (22852) (MNHN), 22 Oct 1985, 3 ovip. (22850) (MNHN), La Grave Les Freaux, *Rosa* sp., 21 Oct 1969, ovip. (22851) (MNHN), 2 ovip. (22853) (MNHN), Villar-d'Arêne, *R*. *spinosissima*, 22 Oct 1969, Remaudière, ♂ (22846) (MNHN), 2 ♂ (22848) (MNHN), Barrême, *Rosa* sp., 07 Nov 1989, Remaudière, ♂ (22847) (MNHN); INDIA: Nainital, *Rosa* sp., 24 May 1969, Narayanan, 4 apt. viv. fem. (BM 1971–428) (BMNH); ITALY: Fenestrelle, *Rosa* sp., 31 Aug 1965, Michel, 4 apt. viv. fem. (22828) (MNHN), Vallombrosa, *Rosa* sp., 30 Aug 1995, O. E. Heie, apt. viv. fem. (8514) (ZMUC); MEXICO: Calpan Huejotzingo, *Rosa* sp., 05 Jun 1983, Muñoz, 4 apt. viv. fem. (22834) (MNHN), Xochimilco (D. F), *Rosa* sp., 27 Dec 1983, Peña, apt. viv. fem. (22856) (MNHN); MOROCCO: Rabat, *Rosa* sp., 19 Apr 1935, Mimeur, 4 apt. viv. fem. (22825) (MNHN), 2 al. viv. fem. (22827) (MNHN); PERU: Cusco, *Rosa* sp., 03 Oct 1989, Bertschinger, 2 apt. viv. fem. (22832) (MNHN); POLAND: Międzyzdroje, *Rosa* sp., 28 Jul 1982, Majchrowicz, 4 apt. viv. fem. (22824) (MNHN); Władysławowo, *Rosa* sp., 09 Jul 1960, Szelegiewicz, 2 al. viv. fem. (R2875, 1342) (ZMAS); PAKISTAN: Murree, *Rosa* sp. (near *brunnonii*), 20 May 1989, Naumann, 2 apt. viv. fem. (22829) (MNHN), 3 apt. viv. fem. (22830) (MNHN), Muree, *Rosa* sp., 01 Jul 1964, v.d. Bosh 6 apt. viv. fem. (BM 1984–340); SWEDEN: Bergkvara, *Dasyphora fruticosa*, 21 Jun 1990, O. E. Heie, al. viv. fem., (8081) (ZMUC); UNITED KINGDOM: Cambridge, *Rosa* sp. (“Rose bush”), 21 Oct 1950, HLGS, 4♂ (BM 1982–492) (BMNH), 4 ovip. (BM 1982–492. 825) (BMNH), Harpenden, *R*. *canina*, 7 Nov 1978, 5 ovip. (BM 1982–794, 7520) (BMNH), Warriner’s Wood Kendal, *R*. *canina*, 11 Nov 1943, F.H. Jacob, ♂ (BM 1984–264, 724) (BMNH); Midhurst, *Rosa* sp., 29 May 1946, Hall, 3 al. viv. fem. (BM 1954–624) (BMNH), Bracknell, *Rosa* sp. (“on roses in garden”), 31 May 1967, Baranyoviys, al. viv. fem. (247/67) (BMNH), Langtoft, *Rosa* sp., 31 May 1944, Doncaster, 9 apt. viv. fem. (BM 1952–23) (BMNH), Notts, *Dasiphora* = *Potentilla*) *fruticosa*, 10 Jul 1977, Martin, 6 apt. viv. fem. (Mr 1) (BMNH), *Rosa* sp., 20 Feb 1976, Martin, 5 apt. viv. fem. (Mr 2) (BMNH), 18 Feb 1978, 4 apt. viv. fem. (Mr 3) (BMNH), Henley, *Fragaria* sp., 26 Jul 1993, V.F.E, apt. viv. fem. (VFE 19503) (BMNH), London, *Rosa* sp., 09 Mar 1975, Martin, apt. viv. fem. (1099) (BMNH); USA: Chelan Co., Manson, Washington, *Dasiphora* = *Potentilla*) *fruticosa*, 21 Jun 2010, A. Jensen, 2 apt. viv. fem. (AJ 4200) (AJ), 2 apt. viv. fem. (AJ 4199) (AJ), 2 apt. viv. fem. (AJ 4201) (AJ), Lakeview, Oregon, *P*. *fructicosa*, 15 Sep 2015, A. Jensen, apt. viv. fem. (AJ 8186) (AJ), 15 Oct 2015, A. Jensen, 3 apt viv. fem. (AJ 2857) (AJ).

**Fundatrix–description** (n = 10)

(Fig 4C; Table 1)

**Colour.** Colour in life: yellowish-green. Colour in mounted specimens: body in general pale or yellowish. Apices of ANT III, ANT IV and ANT V, distal part of tibiae, tarsi, SIPH and cauda light brown to brown (Fig 4C).

**Morphometric characters.** HLS 0.37–0.75 × BD III. ANT 0.28–0.31 × BL and 0.53–0.63 × HW. PT 0.88–1.30 × BASE. Other antennal ratios: ANT V/ANT III about 0.56–1.00, ANT IV/ANT III 0.32–0.43. ANT III with 7–10 setae, ANT IV with 3 setae, ANT V with 3 basal setae. LS III 0.25–0.40 × BD III. URS 0.26–0.50 × ANT III, 0.44–0.53 × ANT V, 0.85–1.15 × BASE and 0.76–0.88 × HT II. HT II 0.33–0.56 × ANT III, 1.05–1.30 × BASE and 0.55–0.62 × ANT V. Dorsal setae on thorax 0.003–0.005 mm long; on abdomen 0.005–0.065 mm long. SIPH 1.65–1.96 × cauda.

**Remarks.** this morph differs from apterous viviparous female by having 5-segmented ANT.

**Apterous viviparous female–re-description** (n = 120)

(Figs 5E–8E; Table 2)

**Colour.** Colour in life: body in general light green to yellow. Colour in mounted specimens: body in general yellow or light brown, with darker distal parts of tibiae and tarsi or with ANT V and ANT V, femora and SIPH darker. Cauda almost always darker than other body parts (Fig 5E).

**Morphometric characters.** Head with median frontal tubercle always well developed, quadrate, usually bearing 2 setae (Fig 6E). Head setae 0.004–0.017 mm long, HLS 0.37–0.85 × BD III. ANT 0.36–0.43 × BL and 0.41–0.56 × HW. PT 1.11–2.00 × BASE. Other antennal ratios: ANT VI/ANTIII 0.89–1.66, ANT V/ANTIII 0.34–0.66, ANT IV/ANT III 0.36–0.64. ANT III with 4–10 setae, ANT IV with 3–7 setae, ANT V with 3–4 setae, ANT VI with 3–4 basal setae. LS III 0.24–0.42 × BD III. URS 0.33–0.83 × ANT III, 0.35–0.50 × ANT VI, 0.83–1.00 × BASE and 0.70–0.95 × HT II. HT II 0.43–0.88 × ANT III, 1.05–1.43 × BASE and 0.42–0.58 ANT VI. Dorsal cuticle mostly in form of oval and often of irregular shape (Fig 7E) Dorsal setae on thorax 0.005–0.010 mm long; on abdomen 0.005–0.027 mm long. SIPH 1.60–2.20 × cauda, tubular, slightly curved. Cauda tongue shaped with broad median part and narrow end (Fig 8E).

**Alate viviparous female–re-description** (n = 21)

(Figs 9D and 10D; Table 3)

**Colour.** Colour in life: unknown. Colour in mounted specimens: head and thorax dark brown to with lighter pronotum. ANT uniformly brown. Fore and hind wings pale yellowish with brown veins. Pterostigma pale brown with dark edges. Legs light brown with lighter proximal parts of femora and darker distal parts of tibiae and tarsi. Abdomen pale with pale brown dorsal sclerotic patch and marginal sclerites. SIPH and cauda pale brown (Fig 9D).

**Morphometric characters.** Head setae 0.007–0.012 mm long, HLS 0.34–0.60 × BD III. ANT 0.53–0.67 × BL and 0.25–0.30 × HW. ANT III with 15–24 secondary rhinaria (Fig 10D). PT 1.30–1.76 × BASE. Other antennal ratios: ANT VI/ANT III 0.71–0.81, ANT V/ANT III 0.40–0.51, ANT IV/ANT III 0.45–0.54. ANT III with 5–14 setae, ANT IV with 4–8 setae, ANT V with 3–5 setae, ANT VI with 3–4 basal setae. LS III 0.34–0.50 × BD III. URS 0.19–0.24 × ANT III, 0.25–0.30 × ANT VI, 0.61–0.75 × BASE and 0.64–0.78 × HT II. HT II 0.26–0.31 × ANT III, 0.91–1.00 × BASE and 0.36–0.39 × ANT VI. Dorsal setae on pronotum about 0.010 mm long; on abdomen 0.007–0.037 mm long. Dorsal abdominal patch formed from fused sclerites on ABD II-VI. SIPH clavate, slightly narrower near base, 1.55–1.82 × cauda which is tongue-shaped.

**Oviparous female–re-description** (n = 19)

(Figs 11D and 12D; Table 4)

**Colour.** Colour in life: unknown. Colour in mounted specimens: head and pronotum sclerotized, brown. ANT brown with lighter ANT I, ANT II and basal part of ANT III. Fore, middle legs and hind tibiae light brown. TIBIAE III light brown with brown swollen part. Abdomen pale with SIPH and cauda light brown (Fig 11D).

**Morphometric characters.** Head with quadrate median frontal tubercle with two setae. Head setae 0.005–0.020 mm long, HLS 0.75–1.00 × BD III. ANT 0.40–0.52 × BL and 0.46–0.50 × HW. PT 1.11–1.43 × BASE. Other antennal ratios: ANT VI/ANT III 0.88–1.38, ANT V/ANT III 0.54–0.62, ANT IV/ANT III 0.43–0.57. LS III 0.30–0.37 × BD III. ANT III with 5–8 setae, ANT IV with 3–5 setae, ANT V with 2–3 setae, ANT VI with 2–3 basal setae. URS 0.36–0.55 × ANT III 0.34–0.41 × ANT VI, 0.83–1.00 × BASE and 0.83–0.94 × HT II. TIBIAE III conspicuously swollen with 20–52 circular and similar in size pseudosensoria, situated in about middle part (Fig 12D). HT II 0.38–0.64 × ANT III, 0.94–1.17 × BASE and 0.39–0.48 × ANT VI. Dorsal setae on thorax 0.005–0.006 mm long; on abdomen 0.005–0.025 mm long. SIPH slightly constricted in the middle and slightly clavate from about middle of length, 1.64–2.13 × cauda, which is broadly tongue-shaped.

**Apterous male–re-description** (n = 10)

(Figs 13C and 14C; Table 5)

**Colour.** Colour in life: unknown. Colour in mounted specimens: Body mostly sclerotized. Head and ANT brown with PT slightly lighter. Thorax, sclerotized parts of abdomen, SIPH and cauda light brown. Femora brown, tibiae light brown with slightly darker distal parts (Fig 13C).

**Morphometric characters.** Head setae 0.008–0.017 mm long, HLS 1.00–1.13 × BD III. ANT (Fig 14C) 0.70–0.85 × BL and 0.30–0.34 × HW. ANT III with 17–30, ANT IV with 2–6, ANT V 2–5 and ANT VI with 0–2 secondary rhinaria. PT 1.52–2.05 × BASE, other antennal ratios: ANT VI/ANT III 0.85–1.07, ANT V/ANT III 0.39–0.50, ANT IV/ANT III 0.32–0.48. ANT III with 7–9 setae, ANT IV with 4–5 setae, ANT V with 3 setae, ANT VI with 3 basal setae. LS III 0.50–0.83 × BD III. URS 0.25–0.31 × ANT III, 0.25–0.34 × ANT VI, 0.73–0.88 × BASE and 0.77–0.93 × HT II. HT II 0.32–0.33 × ANT III, 0.89–0.94 × BASE and 0.31–0.37 × ANT VI. Dorsal setae on thorax 0.005–0.075 mm long; on abdomen 0.005–0.035 mm long. SIPH clavate, constricted at base, 1.90–2.25 × cauda, which is tongue-shaped.

**Diagnosis.** This most common species differs from others in the genus by having a quadrate frontal median tubercle (i.e. equal in length and width) with usually 2 short setae. Apterous viviparous females of *M*. *rosarum* differ from the most similar *M*. *turanica* and *M*. *oezdemirae* **sp. nov.** by (1) having shorter setae on the frontal tubercle– 0.30–0.60 x BD III and (2) no more than 7 setae on genital plate (0.80–1.10 x BD III and more than 8 setae on genital plate in the mentioned species). Alate viviparous females differ from those of *M*. *turanica* by (1) having more secondary rhinaria on ANT III– 15–24 (6–14 in *M*. *turanica*), (2) lower ratio of URS/ANT VI– 0.25–0.30 (0.37–0.42 in *M*. *turanica*). Oviparous females differ from *M*. *turanica* by having 6-segmented ANT (5-segmented in *M*. *turanica*), from *M*. *oezdemirae* **sp. nov.** by less than 52 pseudosensoria on TIBIAE III (more than 61 in *M*. *oezdemirae* **sp. nov.**) and higher ratio of ANT V/ANT III 0.54–0.62 (0.48–0.50 in *M*. *oezdemirae* **sp. nov.**). Males of *M*. *rosarum* differ from those of *M*. *oezdemirae* **sp. nov.** by being apterous and from *M*. *turanica* by 6-segmented ANT and 17–30 secondary rhinaria on ANT III (5-segmented ANT and 7–11 secondary rhinaria on ANT III in *M*. *turanica*).

**Host plants.** The species feeds on numerous wild as well cultivated (especially on rambler) *Rosa* species (for detailed records see [4]). Also, collected from *Dasiphora* = *Potentilla*) *fruticosa* and *Fragaria* sp. [4].

**Distribution.** Very common and almost cosmopolitan species. In most part of Europe, not observed only in Austria, Bosnia & Herzegovina, Croatia, Hungary, Slovenia and southern part of the Balkan peninsula. In North Africa known from Algeria and Morocco. In North America known from Canada, Mexico and USA, in South America in Chile and Peru. In Asia it is known from India, Pakistan and Japan. Also, introduced to New Zealand [4]. The detailed records for countries in Palaearctic are given in Holman [20].

***Myzaphis tianshanica*** Kadyrbekov, 1993

Figs 4D, 5F–8F, 9E and 10E; Tables 1–3

*Myzaphis tianshanica* Kadyrbekov, 1993: 102 [10]

**Material examined. Paratypes**. KAZAKHSTAN: mount Ketmen, 17 km SE of Bol’shoy Aksu, *Rosa albertii*, 24 Jun 1987, R. Kadyrbekov, apt. viv. fem., al. viv. fem., nymph (22803) (MNHN), apt. viv. fem., al. viv. fem. (704) (IZKAS); Karatau, *R*. *albertii*, 29 Jun 1987, 3 fx (736) (IZKAS).

**Fundatrix–re-description** (n = 3)

(Fig 4D; Table 1)

**Colour.** Colour in life: yellow-green [10]. Colour in mounted specimens: body in general pale with yellow distal parts of appendages (ANT V, tarsi and cauda) (Fig 4D).

**Morphometric characters.** HLS 2.50–2.72 × BD III. ANT 0.26–0.32 × BL and 0.53–0.64 × HW. PT 0.77–1.05 × BASE. Other antennal ratios: ANT V/ANT III 0.68–0.74, ANT IV/ANT III 0.41–0.48. ANT III with 3–5 setae, ANT IV with 3–4 setae, ANT V with 2–3 basal setae. LS III 0.45–0.72 × BD III. URS 0.32–0.40 × ANT III, 0.43–0.56 × ANT V, 0.88–1.00 × BASE and 0.80–0.90 × HT II. HT II 0.40–0.45 × ANT III, 1.11–1.16 × BASE and 0.54–0.63 × ANT V. Head setae long, 0.028–0.060. Dorsal setae on thorax long, 0.022–0.060 mm long; on abdomen 0.020–0.075 mm long. SIPH 1.88–2.00 × cauda.

**Remarks:** Besides 5-segmented ANT, this morphs differs from apterous viviparous female by (1) frons almost flat or with very low median frontal tubercle and (2) lower ratio PT/BASE– 0.77–1.05 (1.16–1.20 in apterae).

**Apterous viviparous female–re-description** (n = 2)

(Figs 5F–8F; Table 2)

**Colour.** Colour in life: yellow-green [10]. Colour in mounted specimens: body in general pale, only distal parts of ANT, tibiae and cauda yellow (Fig 5F).

**Morphometric characters**. Head with median frontal tubercle rectangular, wider than longer with 4 long, thickened and pointed setae (Fig 6F). Head setae 0.040–0.080 mm long, HLS 3.40–3.63 × BD III. ANT 0.34–0.39 × BL and 0.45–0.50 × HW. PT 1.16–1.20 × BASE. Other antennal ratios: ANT VI/ANT III 1.21–1.22, ANT V/ANT III 0.65–0.72, ANT IV/ANT III 0.68–0.72. ANT III with 5–6 setae, ANT IV with 2–3 setae, ANT V with 3–6 setae, ANT VI with 3–4 basal setae. LS III 0.54–0.68 × BD III. URS 0.47–0.53 × ANT III, 0.65–0.80 × ANT VI, 0.85–0.94 × BASE and about 0.85 × HT II. HT II 0.55–0.62 × ANT III, 1.00–1.11 × BASE and 0.45–0.51 ANT VI. Dorsal cuticle mostly in form of more or less regular ovals (Fig 7F). Dorsal body setae long to very long, thickened and pointed. Dorsal setae on thorax 0.037–0.070 mm long; on abdomen 0.055–0.090 mm long. SIPH 2.05–2.21 × cauda, straight, not clavate. Cauda tongue-shaped (Fig 8F).

**Alate viviparous female–re-description** (n = 2)

(Figs 9E and 10E; Table 3)

**Colour.** Colour in life: head, ANT, thorax, abdominal patch, SIPH and cauda brown. Unsclerotized part of abdomen and legs yellow [10]. Colour in mounted specimens: head, ANT, thorax and legs brown to light brown. Fore and hind wings pale with light brown veins. Pterostigma pale brown with slightly darker edges. Abdomen pale with light brown dorsal sclerotic patch, SIPH and cauda (Fig 9E).

**Morphometric characters.** Head setae 0.025–0.027 mm long, HLS 2.85–3.35 × BD III. ANT0.57–0.59 × BL and 0.28–0.29 × HW. ANT III with 3–18 secondary rhinaria (Fig 10E). PT 1.26–1.39 × BASE. Other antennal ratios: ANT VI/ANT III 0.80–0.86, ANT V/ANT III 0.61–0.63, ANT IV/ANT III 0.64–0.70. ANT III with 6 setae, ANT IV with 4 setae, ANT V with 3 setae, ANT VI with 4 basal setae. LS III 0.75–0.88 × BD III. URS about 0.22 × ANT III, 0.25–0.27 × ANT VI, 0.57–0.65 × BASE and about 0.83 × HT II. HT II about 0.26 × ANT III, 0.69–0.78 × BASE and 0.30–0.32 × ANT VI. Dorsal setae on pronotum 0.027–0.042 mm long; on abdomen 0.025–0.060 mm long. Dorsal abdominal patch formed from wide fused sclerites on ABD I-VI. SIPH clavate, slightly constricted near base, 1.92–1.96 × cauda which is tongue-shaped.

**Diagnosis.** Among all known species of *Myzaphis*, known morphs of *M*. *tianshanica* differ from the others by having long, conspicuous and pointed setae on head and the dorsal side of thorax and abdomen, as opposed to short, inconspicuous and blunt or slightly capitate setae in other *Myzaphis* species.

**Host plants.** This species was collected from *Rosa albertii*.

**Distribution.** This species is known only from Kazakhstan from Mount Ketmen and Karatau (Tien-shan).

***Myzaphis tuatayae*** Kanturski & Barjadze **sp. nov.**

urn:lsid:zoobank.org:act:3E04C8B0-938E-4303-8B58-C9C03B317B03

Figs 4E and 5G–8G; Tables 1 and 2

**Material examined. Holotype**. TURKEY: Isparta, *Rosa* sp., 08 May 1962, Tuatay, apt. viv. fem. (22805) (MNHN).

**Paratypes**. TURKEY: Isparta, *Rosa* sp., 12 Apr 1962, Tuatay, 2 apt. viv. fem. (22782) (MNHN), apt. viv. fem. (22783) (MNHN), apt. viv. fem. (22784) (MNHN), 4 apt. viv. fem. (22804) (MNHN); Ankara, *Rosa* sp., 12 Apr 1962, Tuatay, 2 fx. (62–3) (NTPPM).

**Fundatrix–description** (n = 2)

(Fig 4E; Table 1)

**Colour.** Colour in life: unknown. Colour in mounted specimens: body and ANT pale, ANT IV pale or with pale brown apex, ANT V pale or pale brown, URS pale brown, tarsi yellow or pale brown (Fig 4E).

**Morphometric characters.** HLS 0.48–0.57 × BD III. ANT 0.28–0.31 × BL and 0.65–0.67 × HW. PT 0.77–1.02 × BASE. Other antennal ratios: ANT V/ANT III 0.66–0.89, ANT IV/ANT III 0.33–0.44. ANT III with 4–8 setae, ANT IV with 3–4 setae, ANT V with 3 basal setae. LS III 0.26–0.33 × BD III. URS 0.35–0.52 × ANT III, 0.53–0.58 × ANT V, 1.03–1.08 × BASE and 0.82–0.85 × HT II. HT II 0.42–0.61 × ANT III, 1.21–1.31 × BASE and 0.64–0.68 × ANT V. Head setae very short, 0.008–0.017. Dorsal setae on thorax very short, 0.009–0.011 mm long; on abdomen 0.008–0.013 mm long. SIPH 1.88–2.00 × cauda.

**Remarks.** Besides 5-segmented ANT, this morphs differs from apterous viviparous female by (1) shorter ANT– 0.61–0.62 (0.66–0.81 in apterae), (2) higher ratio of HW/ANT– 0.65–0.67 (0.46–0.59 in apterae), (3) slightly higher ratio of URS/HT II– 0.82–0.85 (0.72–0.82 in apterae).

**Apterous viviparous female–description** (n = 9)

(Figs 5G–8G; Table 2)

**Colour.** Colour in life: unknown. Colour in mounted specimens: body pale, ANT pale with ANT VI yellow, legs yellowish with yellow to light brown tarsi (Fig 5G).

**Morphometric characters.** Head with almost straight frons without median frontal tubercle, with very slightly rounded median part (Fig 6G). Head setae 0.005–0.14 mm long, HLS 0.43–0.54 × BD III. ANT 0.29–0.39 × BL and 0.46–0.59 × HW. PT 0.77–1.11 × BASE. Other antennal ratios: ANT VI/ANT III 0.73–0.85, ANT V/ANT III 0.38–0.45, ANT IV/ANT III 0.36–0.42. ANT III with 4–9 setae, ANT IV with 1–4 setae, ANT V with 3–4 setae, ANT VI with 3 basal setae. LS III 0.30–0.34 × BD III. URS 0.33–0.45 × ANT III, 0.46–0.53 × ANT VI, 0.94–1.11 × BASE and 0.72–0.82 × HT II. HT II 0.47–0.54 × ANT III, 1.22–1.35 × BASE and 0.63–0.68 ANT VI. Dorsal cuticle mostly in form of ovals and rectangles with wide, rounded edges (Fig 7G) Dorsal setae on thorax 0.005–0.010 mm long; on abdomen 0.006–0.012 mm long. SIPH 1.72–2.20 × cauda, straight, not clavate. Cauda tongue shaped with constricted apex (Fig 8G).

**Etymology.** The authors have the pleasure in naming the new species to honour Dr. Nazife Tuatay, who worked on the Turkish aphid fauna for several decades.

**Diagnosis.** Based on the head without a median frontal tubercle this species is similar to *M*. *juchnevitchae*. Apterous viviparous females of *M*. *tuatayae* Kanturski & Barjadze **sp. nov.** differ from those of *M*. *juchnevitchae* by (1) pigmentation of body dorsum: unpigmented in the new species, (2) frons shape: straight, (3) SIPH shape: straight, tapering towards apex, (4) lower ratio of longest head setae/BD III—0.38–0.45, (5) lower ratio of ANT V/ANT III– 0.38–0.45 (body dorsum pigmented, frons convex, SIPH clavate; longest head setae/BD III 1.25–1.35 x ANT V/ANT III 0.57–0.68 in *M*. *juchnevitchae*).

**Host plants.** This species was collected from an unidentified *Rosa* species.

**Distribution.** The species is known from Isparta (south-western Turkey) and Ankara (Central Turkey).

***Myzaphis turanica*** Nevsky, 1929

Figs 5H–8H, 9F, 10F, 11E, 12E, 13D and 14D; Tables 2–5

*Myzaphis rosarum turanica* Nevsky, 1929: 285 [22].

**Material examined. Lectotype** (present designation). UZBEKISTAN: Tashkent, *Rosa* sp., 03 Jul 1927, collector not mentioned, apt. viv. fem. number 2 in red circle with “L” (2372a) (ZMAS).

**Paralectotypes**. UZBEKISTAN: Tashkent, *Rosa* sp., 03 Jul 1927, collector not mentioned, apt. viv. fem. (2372a) (ZMAS), 2 apt viv. fem. (2372b) (ZMAS).

**Non-type material.** INDIA: Simla, plant unknown, 12 May 1969, Naraynan, 3 apt. viv. fem. (22798) (MNHN), 29 May 1969, L.K. Ghosh, apt. viv. fem. (BM 1984–340) (BMNH), Kashmir, *Rosa webbiana*, 22 Oct 1977, N.D. Rishi, 4 ovip., ♂ (1984-340a) (BMNH), 4 ovip., ♂ (1984-340b) (BMNH), Kasauli, *Rosa* sp., 30 Aug 1980, SD, 4 apt. viv. fem. (22797) (MNHN); ITALY: Catania, Sicilia, *Rosa* sp., 14 May 1980, Barbagallo, 5 apt. viv. fem. (BM 1982–492) (BMNH), 4 al. viv. fem. (BM 1982–492, 234) (BMNH); ISRAEL, Rehovot, *Rosa* sp., 23 Jan 1977, EMZ, 3 apt. viv. fem., al. viv. fem. (506) (BMNH); MONGOLIA: Zaisan at Ulan-Baatar, *Rosa* sp., 10 Jun 1962, Bielawski & Pisarski, 8 apt. viv. fem. (R 2878, 1752) (ZMPA); PORTUGAL–FIRST RECORD: Palmela, *Rosa* sp., 11 Apr 1980, Ilharco, 5 apt. viv. fem. (RLB 2039a) (BMNH), al. viv. fem. (RLB 2039b); TAJIKISTAN: Kondara, *Rosa* sp., 07 Jun 1972, Holman, 4 apt. viv. fem. (22799) (MNHN), Dushanbe, *Rosa* sp., 08 Jun 1972, Holman, 4 apt. viv. fem. (1H01HA) (ZMUC).

**Apterous viviparous female–re-description** (n = 41)

(Figs 5H–8H; Table 2)

**Colour.** Colour in life: unknown. Colour in mounted specimens: body pale to yellowish with slightly darker cauda (Fig 5H).

**Morphometric characters.** Head with median frontal tubercle rectangular, slightly wider than longer, bearing 2–4 setae (Fig 6H). Head setae 0.007–0.032 mm long. HLS 0.66–1.28 × BD III. ANT 0.31–0.41 × BL and 0.42–0.56 × HW. PT 0.86–1.61 × BASE. Other antennal ratios: ANT VI/ANT III 0.86–1.19, ANT V/ANT III 0.44–0.56, ANT IV/ANT III 0.33–0.53. ANT III with 5–8 setae, ANT IV with 2–4 setae, ANT V with 2–4 setae, ANT VI with 3 basal setae. LS III 0.25–0.47 × BD III. URS 0.32–0.51 × ANT III, 0.36–0.50 × ANT VI, 0.85–1.00 × BASE and 0.66–0.90 × HT II. HT II 0.46–0.67 × ANT III, 0.95–1.22 × BASE and 0.46–0.63 ANT VI. Dorsal cuticle mostly in form of ovals or rectangles with rounded edges (Fig 7H). Dorsal setae on thorax 0.007–0.012 mm long; on abdomen 0.010–0.271 mm long. SIPH 1.47–1.96 × cauda, almost straight, slightly clavate. Cauda tongue-shaped (Fig 8H).

**Alate viviparous female–re-description** (n = 6)

(Figs 9F and 10F; Table 3)

**Colour.** Colour in life: unknown. Colour in mounted specimens: head and thorax brown, ANT and femora yellow to light brown, tibiae pale with distal yellow to light brown distal parts and tarsi. Wings pale with brown veins. Pterostigma pale brown. Abdomen pale with abdominal patch, SIPH and cauda yellowish (Fig 9F).

**Morphometric characters.** Head setae 0.010–0.017 mm long, HLS 0.80–1.00 × BD III. ANT 0.43–0.48 × BL and 0.34–0.40 × HW. ANT III with 6–14 secondary rhinaria (Fig 10F). PT 1.10–1.26 × BASE. Other antennal ratios: ANT VI/ANT III 0.62–0.63, ANT V/ANT III 0.29–0.43, ANT IV/ANT III 0.33–0.41. ANT III with 5–7 setae, ANT IV with 3–4 setae, ANT V with 3–4 setae, ANT VI with 3 basal setae. LS III 0.55–0.70 × BD III. URS 0.23–0.26 × ANT III, 0.37–0.42 × ANT VI, 0.84–0.89 × BASE and 0.51–0.77 × HT II. HT II 0.34–0.45 × ANT III, 1.15–1.33 × BASE and 0.55–0.72 × ANT VI. Dorsal setae on pronotum 0.010–0.010 mm long; on abdomen 0.007–0.022 mm long. Dorsal abdominal patch formed from fused spinal sclerites on ABD II-V. SIPH almost straight on whole length, 1.31–1.68 × cauda which is narrow tongue-shaped.

**Oviparous female–re-description** (n = 8)

(Figs 11E and 12E; Table 4)

**Colour.** Colour in life: unknown. Colour in mounted specimens: head, femora, tibiae and SIPH yellow to light brown. ANT V, tarsi and cauda light brown to brown (Fig 11E).

**Morphometric characters.** Head with low, rectangle median tubercle with two setae. Head setae 0.007–0.022 mm long, HLS 0.64–1.40 × BD III. ANT 0.40–0.44 × BL and 0.49–0.57 × HW. PT 1.20–1.37 × BASE. Other antennal ratios: ANT V/ANT III 0.90–1.03, ANT IV/ANT III 0.38–0.47. LS III 0.37–0.47 × BD III. ANT III with 4 setae, ANT IV with 5–7 setae, ANT V with 3 basal setae. URS 0.33–0.37 × ANT III 0.35–0.36 × ANT VI, 0.80–0.87 × BASE and 0.70–0.75 × HT II. TIBIAE III not swollen with 7–20 circular and similar in size pseudosensoria, in the middle of length and on the inner side of tibiae (Fig 12E). HT II 0.47–0.50 × ANT III, 1.06–1.25 × BASE and 0.47–0.52 × ANT V. Dorsal setae on thorax 0.005–0.010 mm long; on abdomen 0.005–0.027 mm long. SIPH only slightly curved in the middle and slightly clavate, 1.78–2.09 × cauda, which is broadly tongue-shaped.

**Apterous male–description** (n = 2)

(Figs 13D and 14D; Table 5)

**Colour.** Colour in life: unknown. Colour in mounted specimens: Body mostly sclerotized. Head, ANT, legs, SIPH and cauda light brown to brown. Sclerotization of thorax and abdomen light brown (Fig 13D).

**Morphometric characters.** Head setae 0.005–0.017 mm long, HLS 1.00–1.06 × BD III. ANT (Fig 14D) 0.61–0.66 × BL and 0.47–0.51 × HW. ANT III with 7–11, ANT IV with 3–5 and ANT V with 1 secondary rhinarium. PT 1.20–1.37 × BASE, other antennal ratios: ANT V/ANT III 0.78–0.86, ANT IV/ANT III about 0.38. ANT III with 9–12 setae, ANT IV with 2–5 setae, ANT V with 2–3 basal setae. LS III 0.58–0.66 × BD III. URS 0.22–0.24 × ANT III, 0.26–0.31 × ANT V, 0.62–0.69 × BASE and 0.58–0.69 × HT II. HT II 0.35–0.38 × ANT III, 1.00–1.06 × BASE and 0.44–0.45 ANT V. Dorsal setae on thorax 0.004–0.005 mm long; on abdomen 0.005–0.020 mm long. SIPH straight, not clavate, 2.05–2.26 × cauda, which is broadly tongue-shaped.

**Diagnosis.** There are three species of the genus *Myzaphis* with well-developed median frontal tubercle. Together with *M*. *oezdemirae* **sp. nov.**, *M*. *turanica* differs from *M*. *rosarum* by having median tubercle wider than long with usually 4 longer setae (quadrate median tubercle bearing 2 short setae in *M*. *rosarum*). From *M*. *oezdemirae* **sp. nov**. apterous viviparous females of *M*. *turanica* differ by (1) lower ratio of URS/ANT III– 0.32–0.51 (0.70–0.87 in *M*. *oezdemirae* **sp. nov.**), (2) lower ratio of HT II/ANT III– 0.42–0.67 (0.85–1.00 in *M*. *oezdemirae* **sp. nov.**) and (3) abdomen pale without two darker longitudinal pleural stripes which are present in *M*. *oezdemirae* **sp. nov.** Oviparous females differ from those of *M*. *oezdemirae* **sp. nov.** by, not swollen TIBIAE III with 7–20 pseudosensoria (TIBIAE III conspicuously swollen with 61–80 pseudosensoria in *M*. *oezdemirae* **sp. nov.**). The males are apterous (alate in *M*. *oezdemirae* **sp. nov.**).

**Host plants.** Like other *Myzaphis* species, *M*. *turanica* feed on several wild *Rosa* species. In the type locality on *R*. *bruniana*. For detailed records see Blackman & Eastop (2017).

**Distribution.** In Europe, mostly in Mediterranean region (France, Spain, Italy-Sicilia, and Portugal–FIRST RECORD); the record in Sweden needs conformation. Also in the Middle East (Israel), Central Asia (Kazakhstan, Kirgistan, Tajikistan, Uzbekistan) and other parts of Asia (India and Mongolia). The detailed records for countries in Palaearctic are given in Holman [20].

#### Remarks on the taxonomic status of *M*. *komatsubarae* Shinji

From the very short and general description, “Characteristics: Body green to pale. Antennae infuscated throughout, III longer than IV and V taken together with about 19 subcircular sensoria, flagellum of VI about as long as base. Rostrum black throughout. Siphunculi black, cauda infuscated or black” [6], only little can be deduced. From the information about the secondary rhinaria on ANT III it is likely that the species was described from an alate viviparous female. Even if the antennae of alate some species of Myzaphis could be more or less brown to dark, neither the SIPH or cauda are dark or black. Also, the length of ANT III in contrast to ANT IV and V is a variable character not only among aphid genera but also among the species of Myzaphis, but in all alate viviparous females it is more or less slightly longer that the length of the two next ones.

It is impossible to establish the identity of the species as well as its generic affinity. Its hostplant being *Sorbus commixta* hints that it could be a species of *Dysaphis* Börner, 1931 [4].

Due to the absence of any available material, drawings or good description to evaluate this species, we consider *Myzaphis komatsubarae* Shinji, 1922 as a nomen dubium.

***Ericaphis* Börner, 1939**

*Ericaphis* Börner, 1939: 80 [23].

Type species: *Myzaphis ericae* Börner, 1933, by original designation.

***Ericaphis avariolosa*** (David, Rajasingh & Narayanan, 1970) **comb. nov.**

Figs 5I–8I; Table 2

*Myzaphis avariolosa* David, Rajasingh & Narayanan, 1970 [8]: 397.

**Material examined. Paratype**. INDIA: Manali, *Rosa macrophylla*, 11 Jun 1968, Rajasingh & Naraynan, apt. viv. fem. (BM 1984–340) (BMNH).

**Apterous viviparous female–re-description** (n = 1)

(Figs 5I–8I; Table 2)

**Colour.** Colour in life: body green, ANT, legs and SIPH pale [8]. Colour in mounted specimens: head, ANT, legs, SIPH and cauda yellow. Thorax and abdomen pale (Fig 5I).

**Morphometric characters.** body spindle shaped with long ANT and legs. Head without median frontal tubercle with low but well developed and evident ANT tubercles. Dorsal and ventral side of head, especially near the ANT tubercles with numerous well-developed spicules (Fig 6I). Head setae very short, 0.005–0.007 mm long and blunt. HLS 0.28–0.30 × BD III. ANT 6-segmented, 0.63–0.64 × BL, 0.27–0.28 × HW, without secondary rhinaria. ANT III longest, ANT IV slightly longer than ANT V which is shorter than ANT VI. PT 1.69–2.00 × BASE. Other antennal ratios: ANT VI/ANT III 0.78–1.09, ANT V/ANT III 0.44–0.47, ANT IV/ANT III 0.23–0.53. ANT setae very short and inconspicuous, ANT III with 9–10 setae, ANT IV with 4–5 setae, ANT V with 3–4 setae, ANT VI with 3 basal and 4 apical setae. LS III 0.20–0.27 × BD III. Rostrum reaching middle coxae, URS about 0.25 × ANT III, 0.27 × ANT VI, 0.73–0.76 × BASE and 0.86–0.90 × HT II with 4 accessory setae. HT I with 5-5-5 ventral setae. HT II 0.27–0.28 × ANT III, 0.80–0.88 × BASE and 0.30–0.31 ANT VI. Dorsum not sclerotized, membranous without any sclerotized markings, smooth, never rugose or corrugated (Fig 7I). Dorsal setae on thorax 0.004–0.005 mm long; on abdomen 0.004–0.037 mm long, inconspicuous with blunt apices. SIPH 2.21–2.47 × cauda, almost straight, very slightly swollen in about ¾ of their length, imbricated. Cauda tongue-shaped with 4 setae (Fig 8I).

**Diagnosis.** *Ericaphis avariolosa* **comb. nov.** differs from all known species of the genus *Myzaphis* in development and shape of frons and ANT tubercles. This species is characterized by completely undeveloped median frontal tubercle and prominent, steep-sided ANT tubercles (completely undeveloped in remaining *Myzaphis* species, also these with low or undeveloped frontal tubercle, but then the frons often rounded). There is also one important difference between *E*. *avariolosa* **comb. nov.** and all known *Myzaphis* species–head surface with prominent, numerous spicules also on the ANT tubercles (head completely smooth in *Myzaphis*). *Ericaphis* species are known to have membranous or more or less sclerotic dorsal side of body, but like *E*. *avariolosa* **comb. nov.** their cuticle never forms any pattern, is not rugose nor wrinkled, which characterizes the members of *Myzaphis*. Other differences between this species and *Myzaphis* include: clearly longer femora and tibiae relative to body length, ANT longer than half of body length– 0.63–0.64 x BL (0.29–0.43 in *Myzaphis*). HW 0.27–0.28 × ANT only (0.41–67 in *Myzaphis*), longer SIPH– 2.21–2.31 x cauda (1.26–2.21 in *Myzaphis*), longer ANT III– 0.32–0.38 (0.09–0.26 in *Myzaphis*), cauda with only 4 setae (6–7 setae in *Myzaphis*).

We think that this species has affinity to *Ericaphis* Börner, 1939 based on combinations of the following characters: (1) well developed antennal tubercles; (2) presence of numerous spicules on the head and ANT tubercles; (3) absence of numerous rounded depressions on the dorsum of abdomen; (4) number of setae on cauda: 4; (5) presence of 5 setae on the first tarsal segments; (6) presence of long appendages; (7) association with *Rosa* spp.

From known *Ericaphis* species, which are known to feed on *Rosa* spp.–*E*. *fimbriata* (Richards, 1959) and *E*. *wakibae* (Hottes, 1934) this species differs by HT I with 5 ventral setae; from *E*. *fimbriata* additionally it differs by longer SIPH which are 2.21–2.47 x cauda (1.50–1.90 in *E*. *fimbriata*); from *E*. *wakibae* the species additionally differs by shorter URS which is 0.86–0.90 x HT II (1.10–1.40 x HT II in *E*. *wakibae*).

**Host plants.** This species lives on *Rosa macrophylla*.

**Distribution.** So far known only from the type locality in Manali, Himachal Pradesh, India.

#### Redefinition of the genus *Myzaphis* van der Goot, 1913

Type species: *Aphis rosarum* Kaltenbach, 1843, by original designation

**Diagnosis**. From other similar genera of Macrosiphini (especially *Chaetosiphon* and *Longicaudus*) this genus can be distinguished by: small, spindle-shaped, or oval body with short appendages. Head with weakly developed antennal tubercles. ANT only about half of body length, without secondary rhinaria in apterae. Alatae have secondary rhinaria on ANT III only, or on ANT III-IV. Dorsal body setae are blunt and somewhat capitate. First tarsal segments all have 5 setae. The dorsum of the aptera always ornamented with numerous small rounded depressions. Alatae have dusky or dark sclerotic markings, often forming a central dorsal abdominal patch. Spiracular apertures are partly covered by opercula. SIPH are elongated, cylindrical for much of their length with the distal part often curved outwards and slightly swollen, and with a small, but distinct flange. The cauda is tongue-shaped or elongated triangular. Diagnosis was suggested by Blackman [18] with minor modification made by the current authors.

**Remarks.** Shape of median tubercle is removed from the diagnostic characters of the genus *Myzaphis* van der Goot, 1913 given in Blackman [18], because *Myzaphis juchnevitschae* Kadyrbekov, 1993 and *M*. *tuatayae* Kanturski & Barjadze **sp. nov.** do not have a median frontal tubercle. The dorsum of the aptera always ornamented with numerous small rounded depressions in all eight described species. We removed information on “sclerotic and wrinkled” dorsum of apterous females because it is characteristic only for *Myzaphis canadensis* Richards, 1963. Besides, *Myzaphis canadensis* is distinct from *Myzaphis* as here defined, based on differences in the first tarsal chaetotaxy (it has 2-2-2 setae, while all remaining *Myzaphis* species have 5-5-5 setae) and presence of secondary rhinaria on ANT V in alatae; therefore a new genus *Richardsaphis* Kanturski & Barjadze **gen. nov.** is erected. Besides, *Myzaphis avariolosa* David, Rajasingh & Narayanan, 1971 is distinct from *Myzaphis* as here defined based on the following: (1) spiculose ventral side of head, especially frons and area of ANT I and compound eyes (Fig 6I), while spicules absent on head ventral side in all remaining *Myzaphis* species (Fig 6A–6H); (2) presence of well developed, steep–sided antennal tubercles (Fig 6I), while all remaining *Myzaphis* species have low antennal tubercles (Fig 6A–6H); (3) presence of long appendages (Fig 5I), while all remaining *Myzaphis* species have short appendages (Fig 5A–5H). On the basis of these features *M*. *avariolosa* is transferred to the genus *Ericaphis* and new combination for this species is suggested. *Myzaphis komatsubarae* was recognized as”nomen dubium”, because type material is lost (M. Miyazaki, personal communication) and the description is inadequate for recognition of this taxon.

***Richardsaphis* Kanturski & Barjadze gen. nov.**

urn:lsid:zoobank.org:act:87EE43C1-8183-4F7E-85E4-25B57B204BC6

Type species: *Richardsaphis canadensis* **comb. nov.** = *Myzaphis canadensis* Richards, 1963, by original designation.

**Description. Apterous viviparous female**. Small, spindle-shaped aphids with rather short appendages. ANT 6-segmented, only 1/3 of body length, without secondary rhinaria. PT shorter than BASE. The head has low antennal tubercles and rounded median tubercle. Rostrum short, not reaching to middle coxae. URS oblong triangular with blunt apices. First tarsal segments all have 2 ventral setae. Dorsal body setae are blunt and somewhat capitate. The dorsum is sclerotic and wrinkled. Subgenital and anal plates and cauda with spinulose imbrications. SIPH are rather long, with the distal part often slightly swollen, and with a small, but distinct flange. The cauda is tongue-shaped.

**Alate viviparous female**. It has secondary rhinaria on antennal segments III-IV and often V, and dusky sclerotic markings on abdomen or a central dorsal abdominal patch. Presence of central dorsal abdominal patch in alate viviparous females was not mentioned in the original description [7], but investigation of type material (2 alate viviparous females) shows us that they have a pale abdominal patch.

**Etymology.** The generic name *Richardsaphis* is of feminine gender and the authors have the pleasure in naming the new genus to honor Dr. W.R. Richards, who was an outstanding aphid taxonomist at the Canadian National Collection of Insects, Ottawa, Canada and “*aphis”* (plant louse).

**Diagnosis.** New genus resembles the genus *Myzaphis* by shape of antennal tubercles, siphunculi and cauda, absence of secondary rhinaria in apterae and presence of central dorsal abdominal patch on the abdomen in alatae. Both genera live on *Rosa* spp. They differ from each other by: (1) first tarsal chaetotaxy. *Richardsaphis* **gen. nov.** has 2:2:2, while it is 5:5:5 in *Myzaphis*; (2) dorsum of abdomen in apterae: sclerotic and wrinkled without numerous small rounded depressions in *Richardsaphis* **gen. nov.**, while numerous, small, rounded depressions always present in *Myzaphis;* (3) presence/absence of secondary rhinaria on ANT V in alate females: secondary rhinaria often present on ANT V in *Richardsaphis* **gen. nov.**, while they are always absent on ANT V in *Myzaphis*.

In the tribe Macrosiphini 2:2:2 first tarsal chaetotaxy is a characteristic feature for the genera *Hydaphias* Börner, 1930 (five species), *Pseudacaudella* Börner, 1950 (one species) and *Staegeriella* Hille Ris Lambers, 1947 (two species), for the subgenus *Myzus* (*Galiobium)* Börner, 1933 (two species), for the species—*Brachycaudus (Thuleaphis) acaudatus* (Hille Ris Lambers, 1960), *Cryptosiphum mordvilkoi* Ivanovskaja, 1960, and *Micromyzella kathleenae* Remaudière, 1985.

***Richardsaphis canadensis*** (Richards, 1963) comb. nov.

Figs 15–18; Table 6

*Myzaphis canadensis* Richards, 1963: 684 [7].

**Fig 15:**
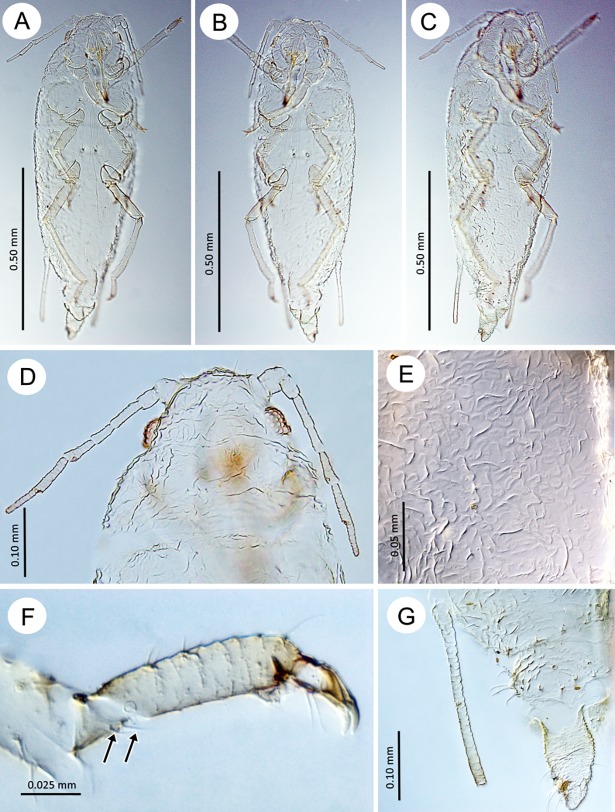
Apterous viviparous female characters of *Richardsaphis canadensis* comb. nov. (A-C) general view. (D) head ANT and pronotum. (E) dorsal abdominal sclerotization pattern. (F) hind tarsus with two ventral setae on HT I (arrows). (G) posterior part of abdomen.

**Fig 16:**
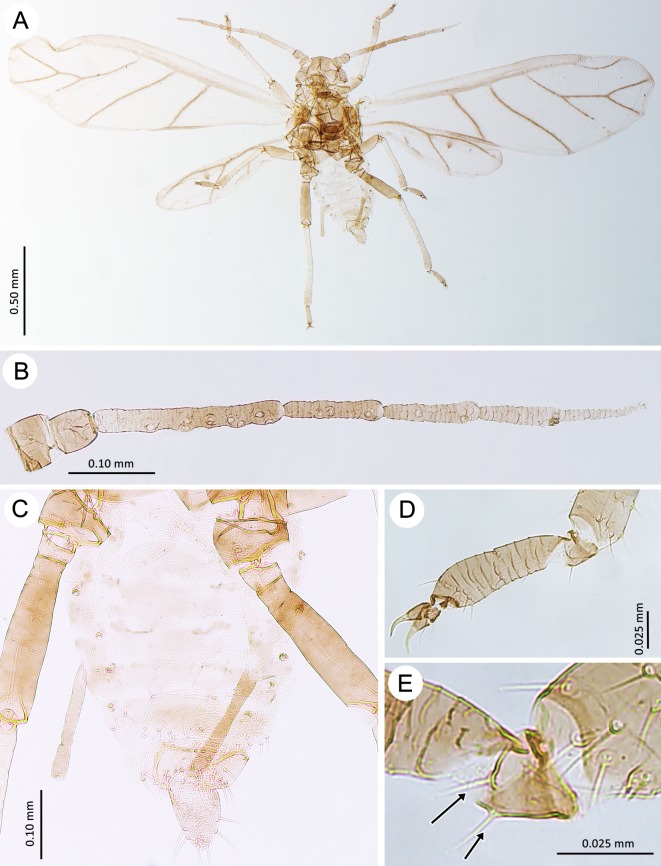
Alate viviparous female characters of *Richardsaphis canadensis* comb. nov. (A) general view. (B) ANT. (C) adomen. (D) hind tarsus. (E) HT I with two dorsal setae (arrows).

**Fig 17:**
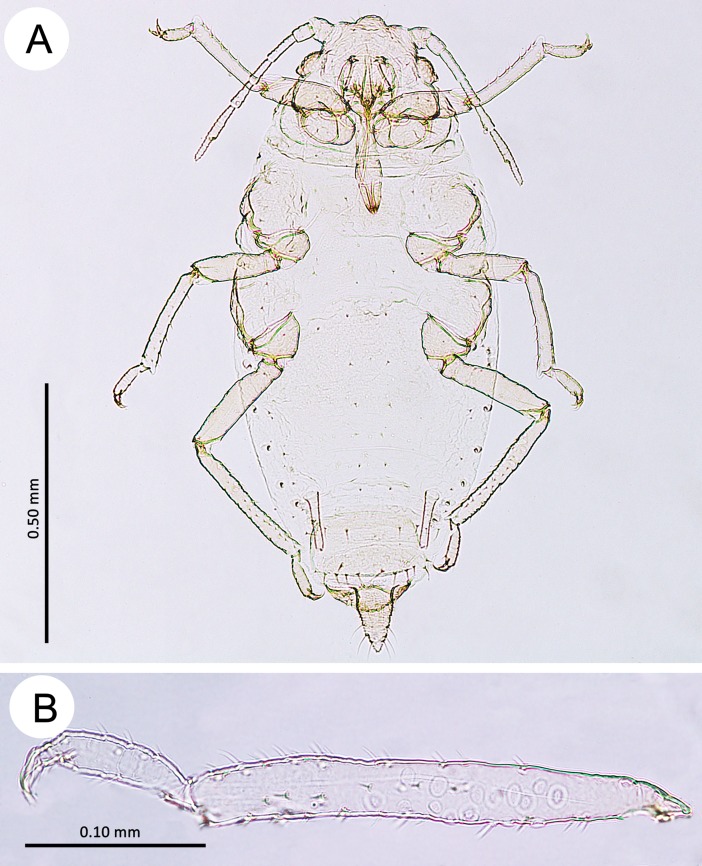
Oviparous female characters of *Richardsaphis canadensis* comb. nov. (A) general view. (B) hind tibia with pseudosensoria.

**Fig 18:**
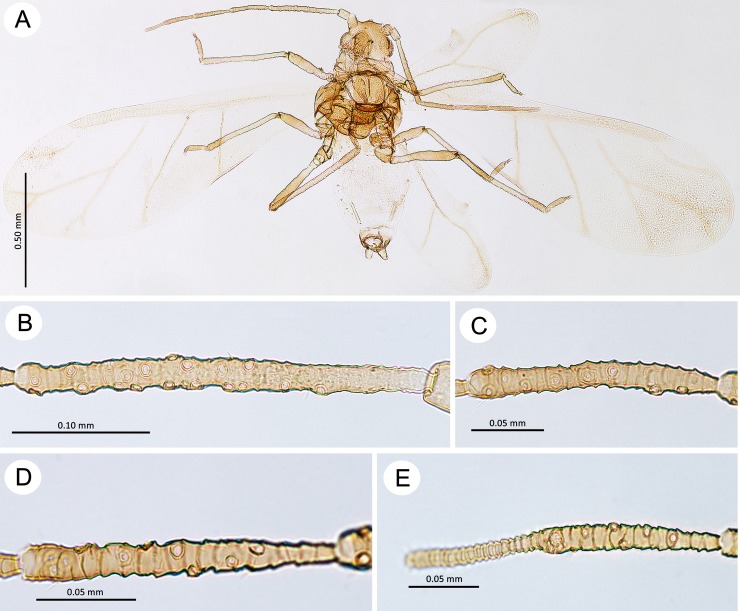
Alate male characters of *Richardsaphis canadensis* comb. nov. (A) general view. (B) ANT III. (C) ANT IV. (D) ANT V. (E) ANT VI with secondary rhinaria.

**Table 6:** Measurements of known morphs of *Richarsaphis canadensis* comb. nov.

Character	Apterous viviparous female n = 5	Alate viviparous female n = 2	Oviparous female n = 3	Male n = 1
**BL**	1.35–1.40	1.04–1.13	1.15–1.22	1.00
**HW**	0.25–0.31	0.24–0.27	0.24–0.25	0.22
**ANT**	0.36–0.40	0.66–0.80	0.36–0.43	0.89–0.92
**ANT III**	0.06–0.07	0.20–0.26	0.06–0.08	0.29
**ANT IV**	0.05	0.10–0.13	0.05–0.08	0.15–0.16
**ANT V**	0.05–0.06	0.10	0.05–0.06	0.14–0.15
**ANT VI**	0.13–0.14	0.19–0.24	0.12	0.20–0.21
**BASE**	0.07–0.08	0.08–0.10	0.06–0.07	0.10–0.11
**PT**	0.05–0.06	0.11–0.14	0.04–0.06	0.09–0.11
**URS**	0.07	0.06–0.07	0.06	0.07
**III FEMUR**	0.19–0.20	0.23–0.25	0.18	0.25
**III TIBIA**	0.32–0.33	0.44–0.47	0.27	0.44–0.45
**HT I**	0.01–0.03	0.02	0.02	0.02
**HT II**	0.08–0.09	0.08–0.10	0.07	0.09
**SIPH**	0.28–0.29	0.15–0.18	0.10–0.11	0.09–0.10
**CAUDA**	0.15–0.17	0.09–0.10	0.10–0.12	0.07

**Material examined. Holotype**. CANADA: Gore Bay (Manitoulin Island, Ontario), *Potentilla fruticosa*, 10 Jul 1961, Richards, apt. viv. fem. (8189) (CNC).

*Paratypes*. CANADA: Gore Bay (Manitoulin Island, Ontario), *Dasiphora* = *Potentilla*) *fruticosa*, 10 Jul 1961, Richards, 4 apt. viv. fem., al. viv. fem. (8189) (CNC), al. viv. fem (22806) (MNHN).

**Non-type material.** USA, FIRST RECORD: Sandoval County, New Mexico, *Dasiphora* = *Potentilla*) *fruticosa*, 23 Sep 2010, A. Jensen, ovip., ♂ (AJ 4582) (AJ), 2 ovip. (AJ 4581) (AJ).

**Apterous viviparous female–re-description** (n = 5)

(Fig 15; Table 6)

**Colour.** Colour in life: body in general light green to yellow [7]. Colour in mounted specimens: body colourless except sclerotized parts of mouthparts, distal parts of ANT VI and tarsi which are pale yellow (Fig 15A–15C).

**Morphometric characters.** Head with low, rounded median frontal tubercles (Fig 15D). Head setae 0.006–0.012 mm long, HLS 0.52–0.62 × BD III. ANT 0.26–0.28 × BL and 0.69–0.76 × HW. PT 0.73–0.78 × BASE. Other antennal ratios: ANT VI/ANT III 2.05–2.20, ANT V/ANT III 0.88–0.94, ANT IV/ANT III 0.71–0.80. ANT III with 6–10 setae, ANT IV with 8–9 setae, ANT V with 7–9 setae, ANT VI with 7–8 basal setae. LS III 0.37–0.43 × BD III. Rostrum reaching between fore and middle coxae, URS 1.08–1.12 × ANT III, 0.51–0.52 × ANT VI, 0.88–0.93 × BASE and 0.82–0.85 × HT II, with 4–5 accessory setae. HT II 1.26–1.37 × ANT III and, 1.07–1.09 × BASE 0.61–0.62 ANT VI. Dorsal cuticle membranous, wrinkled or creased, never in form of squares, rectangles or ovals (Fig 15E). Dorsal setae very short, rigid and blunt, on thorax 0.005–0.013 mm long; on abdomen 0.008–0.043 mm long. HT I with 2 ventral setae (Fig 15F). SIPH 1.70–1.81 × cauda, slender, slightly tapering or slightly clavate. Cauda tongue-shaped with broad median part, slightly constricted at base and apex (Fig 15G).

**Alate viviparous female–re-description** (n = 2)

(Fig 16; Table 6)

**Colour.** Colour in life: abdomen pale green, other body parts brown to black [7]. Colour in mounted specimens: head thorax and femora light brown. ANT, distal parts of tibiae, tarsi, abdomen, SIPH and cauda yellowish to pale brown. Wings pale with brown veins. Pterostigma pale (Fig 16A).

**Morphometric characters.** HW 0.33–0.35 × ANT. Head setae 0.005–0.023 mm long, HLS 0.30–0.80 × BD III. ANT 0.64–0.71 × BL. ANT III with 6–13, ANT IV with 1–4 and ANT V with 1–3 secondary rhinaria (Fig 16B). PT 1.25–1.40 × BASE. Other antennal ratios: ANT VI/ANT III 0.92–0.99, ANT V/ANT III 0.38–0.50, ANT IV/ANT III about 0.50. ANT III with 7–9 setae, ANT IV with 6–10 setae, ANT V with 5–9 setae, ANT VI with 6–8 basal setae. LS III 0.34–0.36 × BD III. URS 0.28–0.34 × ANT III, 0.31–0.34 × ANT VI, 0.75–0.78 × BASE and 0.73–0.78 × HT II with 4 accessory setae. HT II 0.39–0.44 × ANT III, 1.00–1.02 × BASE and 0.42–0.44 × ANT VI. Dorsal setae on pronotum about 0.008–0.012 mm long; on abdomen 0.008–0.042 mm long. Dorsal abdominal sclerotization poorly developed in form of spino-pleural, separate cross bars on ABD II-VII (Fig 16C). HT I with 2 ventral setae (Fig 16D and 16E). SIPH slightly clavate at apex, 1.53–1.66 × cauda which is broadly tongue-shaped.

**Oviparous female–description** (n = 2)

(Fig 17; Table 6)

**Colour.** Colour in life: yellow-pink. Colour in mounted specimens: pale, only more sclerotized body parts yellowish (Fig 17A).

**Morphometric characters.** Head with prominent, rounded median frontal tubercles with four setae. Head setae 0.006–0.022 mm long, HLS about 1.33 × BD III. ANT short, 0.29–0.36 × BL and 0.58–0.69 × HW. PT 0.60–0.92 × BASE. Other antennal ratios: ANT VI/ANT III 1.47–2.00, ANT V/ANT III 0.76–0.91, ANT IV/ANT III 0.66–0.94. LS III 0.24–0.33 × BD III. ANT with only few setae, ANT III with 0–1 setae, ANT IV with 1 setae, ANT V with 2 setae, ANT VI with 1–2 basal setae. Rostrum reaching from between fore and middle coxae to middle coxae, URS 0.76–1.00 × ANT III 0.50–0.52 × ANT VI, 0.80–1.00 × BASE and 0.77–0.86 × HT II. Hind tibiae normal shaped, not swollen with 11–19 circular or egg-shaped different in size pseudosensoria, situated in about middle part of tibiae (Fig 17B). HT II 0.88–1.28 × ANT III, 1.02–1.15 × BASE and 0.60–0.64 × ANT VI. Dorsal setae on thorax 0.00–0.012 mm long; on abdomen 0.007–0.050 mm long. SIPH very short and slender, 0.88–1.00 × cauda, which is broadly tongue-shaped with slightly constricted basal part.

**Alate male–description** (n = 1)

(Fig 18; Table 6)

**Colour.** Colour in life: yellow-pink. Colour in mounted specimens: head, thorax and ANT yellow. Legs yellow with pale tibiae with darker distal parts. Wings pale to yellowish with yellowish veins. Pterostigma pale. SIPH and cauda pale. (Fig 18A).

**Morphometric characters.** Head setae 0.007–0.017 mm long, HLS 1.30–1.41 × BD III. ANT long, 0.89–0.92 × BL and 0.23–0.24 × HW. ANT III with 21 (Fig 18B), ANT IV with 7–9 (Fig 18C), ANT V 10-11(Fig 18D) and ANT VI with 7 secondary rhinaria (Fig 18E). PT 0.78–1.10 × BASE, other antennal ratios: ANT VI/ANT III 0.69–0.72, ANT V/ANT III 0.48–0.52, ANT IV/ANT III 0.51–0.54. ANT III with 4–6 setae, ANT IV with 2–3 setae, ANT V with 3 setae, ANT VI with 1 basal setae. LS III 0.57–0.62 × BD III. URS 0.23–0.24 × ANT III, 0.45–0.50 × ANT VI, 0.60–0.70 × BASE and about 0.77 × HT II. HT II 0.30–0.31 × ANT III, 0.78–090 × BASE and 0.42–0.43 ANT VI. Dorsal setae on thorax 0.001–0.010 mm long; on abdomen 0.005–0.025 mm long. SIPH short, only very slightly clavate at apex, 1.20–1.33 × cauda, which is tongue-shaped.

**Host plants.** This species feed on *Dasiphora fruticosa* = *Potentilla fruticosa*).

**Distribution.** This species was previously known only from the type locality in Canada but was recently also collected in the Jemez Mountains in New Mexico, USA.
